# Efficacy and synergy of live-attenuated and inactivated influenza vaccines in young chickens

**DOI:** 10.1371/journal.pone.0195285

**Published:** 2018-04-06

**Authors:** Hyesun Jang, Mohamed Elaish, Mahesh KC, Michael C. Abundo, Amir Ghorbani, John M. Ngunjiri, Chang-Won Lee

**Affiliations:** 1 Food Animal Health Research Program, Ohio Agricultural Research and Development Center, The Ohio State University, Wooster, Ohio, United States of America; 2 Department of Veterinary Preventive Medicine, College of Veterinary Medicine, The Ohio State University, Columbus, Ohio, United States of America; Icahn School of Medicine at Mount Sinai, UNITED STATES

## Abstract

Outbreaks of novel highly pathogenic avian influenza viruses have been reported in poultry species in the United States since 2014. These outbreaks have proven the limitations of biosecurity control programs, and new tools are needed to reinforce the current avian influenza control arsenal. Some enzootic countries have implemented inactivated influenza vaccine (IIV) in their control programs, but there are serious concerns that a long-term use of IIV without eradication may result in the selection of novel antigenically divergent strains. A broadly protective vaccine is needed, such as live-attenuated influenza vaccine (LAIV). We showed in our previous studies that pc4-LAIV (a variant that encodes a C-terminally truncated NS1 protein) can provide significant protection against heterologous challenge virus in chickens vaccinated at 2–4 weeks of age through upregulation of innate and adaptive immune responses. The current study was conducted to compare the performances of pc4-LAIV and IIV in young chickens vaccinated at 1 day of age. A single dose of pc4-LAIV was able to induce stronger innate and mucosal IgA responses and protect young immunologically immature chickens better than a single dose of IIV. Most importantly, when 1-day-old chickens were intranasally primed with pc4-LAIV and subcutaneously boosted with IIV three weeks later, they showed a rapid, robust, and highly cross-reactive serum antibody response and a high level of mucosal IgA antibody response. This vaccination regimen warrants further optimization to increase its range of protection.

## Introduction

Avian influenza (AI) is a major zoonotic viral disease that causes significant adverse impacts on poultry production, the global trade, and public health [1–4]. Despite decades of research and control efforts, the incidences and severity of AI outbreaks have not been alleviated but rather increased [5, 6]. The strategies currently being employed to control AI are focused on prevention of virus introduction by maintaining strict biosecurity procedures [7–10]. However, the current biosecurity systems have repeatedly failed to protect poultry farms from introduction of novel strains that continue to cause major outbreaks [11, 12]. To overcome this challenge, some countries have incorporated vaccination with inactivated influenza vaccine (IIV) in their control programs [10, 13]. The United States has been using ‘stamping out’ as the primary control strategy, but the occurrence of recent highly pathogenic avian influenza (HPAI) outbreaks [13, 14] further proves the limitations of stamping out in combination with biosecurity control. For this reason, vaccination is being more seriously considered to be added to the country’s AI control arsenal. The IIV is currently in use in countries like Bangladesh, China, Egypt, Indonesia and Viet Nam where HPAI viruses are endemic [7]. Unfortunately, although IIV can provide good protection from homologous field strains [13], it is weak against heterologous strains that arise from random genetic mutations and not protective against heterosubtypic strains [9, 15, 16]. In addition to having a narrow range of protection, there are serious concerns that a long-term use of IIV without eradication of heterologous (mismatched) strains may result in the selection of antigenically divergent strains [17].

Live vaccines have numerous advantages over IIV that can be exploited further to develop new broadly protective vaccines and vaccination regimens. Since a live agent can mimic the natural infection, it can elicit a broad range of immune responses including humoral, cell-mediated and mucosal immunity [18–20]. Importantly, live vaccine can be directly administered on the mucosal surface by spray or through drinking water, which not only elicits local mucosal immunity but also significantly reduces the cost of mass administration [19, 21].

Currently, the only live influenza vaccines available for use in the poultry industry are live viral-vectored vaccines [10, 16]. Live viral-vectored vaccines can induce a broader range of protection compared to IIV, but they have several shortcomings including reduced efficacy due to preexisting immunity to the viral vector and the difficulty of expressing two or more influenza virus proteins in the same vector [16, 22, 23]. Live-attenuated influenza vaccine (LAIV) is an excellent alternative to the vectored vaccine or IIV since it contains all proteins that are naturally found in influenza virus particles [19, 24]. In humans, LAIV has been used for more than a decade and it has been reported to protect young individuals better than IIV [25–27]. Importantly, recent studies showed that LAIV can pre-sensitize the population and, subsequently, synergistically boost the efficacy of IIV [28, 29]. It should be noted that the use of LAIV in poultry requires strict safety standards due to concerns about the possibility that wild type strains may circulate in domestic poultry without apparent clinical symptoms, undergo genetic reassortment with the vaccine virus, and produce novel virulent strains [30, 31]. An ideal poultry LAIV should not be able to revert to wild type virus or undergo reassortment with field strains.

One of the promising candidates for poultry LAIV is pc4-LAIV, an influenza virus mutant that expresses a C-terminally truncated nonstructural protein 1 (NS1 protein) due to a large internal deletion (190 nt) in the NS gene segment, that caused a shift in the NS1 open reading frame and a premature stop codon [19]. Truncation of 137 amino acids from NS1 means that pc4-LAIV cannot easily revert to the wild type phenotype. In two independent vaccination studies, we demonstrated that pc4-LAIV is attenuated and does not transmit from chicken to chicken [19, 32]. Interestingly, even though pc4-LAIV did not replicate efficiently in young birds, it was able to induce robust adaptive immune responses and provide protection against heterologous virus infection [19, 32]. Available evidence suggests that the high level of immunogenicity of pc4-LAIV, despite its poor replication in chickens, is due to the deficiency of its NS1 in antagonizing the induction and signaling pathways of type I interferon (IFN) [32–34].

Influenza NS1 is known to block type I IFN response to create an environment that is favorable for virus replication and truncation of NS1 can lead to an increase in type I IFN production and attenuation of the virus in animals [20, 34]. We previously showed that infection with pc4-LAIV results in induction of high levels of type I IFN production in avian cells *in vitro* [33]. Although we were unable to detect IFN-α/β gene upregulation in vaccinated birds, there were strong correlations between interferon stimulated gene (ISG) upregulation, acceleration of serum antibody response, and induction of a high level of protection against heterologous virus in 4-week-old chickens [32]. In the same study, oral administration of recombinant chicken IFN-α was shown to mimic pc4-LAIV by inducing a rapid serum antibody response to IIV vaccination and an enhanced ISG response suggesting that pc4-LAIV efficacy is dependent on stimulation of the type I IFN system in the vaccinated host [32]. A similar effect was reported by Meng *et al* [35] in a study where oral treatment with recombinant chicken IFN-α resulted in induction of ISG transcription and inhibition of H9N2 influenza virus replication in 7 and 33-day-old chickens [35].

Live vaccines are more efficacious than inactivated vaccines in young, immunologically immature, chickens [36]. We have demonstrated that single vaccination with pc4-LAIV was highly immunogenic and protective in 2–4 weeks-old chickens [19, 32]. Based on our previous findings, the current study was conducted to compare the performances of pc4-LAIV (hereinafter referred to as LAIV) and IIV in young chickens vaccinated at 1 day of age. Although the performance of LAIV was better than IIV, and adaptive immune responses appeared to depend on the type of vaccine, neither of the vaccines could provide full protection against heterologous challenge virus. Subsequently, we designed and evaluated a vaccination regimen in which birds were primed with LAIV and boost-vaccinated with IIV at 1 day and 3 weeks of age, respectively. Not only did the prime-boost induce robust mucosal antibody responses, it also led to acceleration of seroconversion, synergistic increase of serum antibody titers, an increase in antibody cross-reactivity with heterologous antigen, and full protection from heterologous challenge virus.

## Materials and methods

### Animals and ethics statement

All experimental animals were handled as previously described [32]. The animals were maintained, vaccinated, challenged and euthanized in accordance with protocol #2009AG0002 approved by The Ohio State University Institutional Animal Care and Use Committee (IACUC). This protocol complies with the U.S Animal Welfare Act, Guide for Care and Use of Laboratory Animals and Public Health Service Policy on Humane Care and Use of Laboratory Animals. The Ohio State University is accredited by the Association for the Assessment and Accreditation of Laboratory Animal Care International (AAALAC). White leghorn chickens were obtained from our institutional (Food Animal Health Research Program, Wooster, OH) specific pathogen free (SPF) flock. The chickens were housed in a BSL2 facility with forced air ventilation and adequate air exchanges to prevent ammonia build up. Air entering or leaving the facility is HEPA filtered. The birds were kept in large cages (2592 sq. inch) before infection and transferred to Model 934–1 isolators (900 sq. inch) (Federal Designs Inc., Comer, GA). The number of birds in each cage was calculated based on age and the space available after subtracting the space occupied by the feeder and the watering system. Room and isolator temperatures were maintained at 25±3°C. Birds had *ad libitum* access to feed and water. The wellbeing and health status of the animals was monitored twice daily throughout the experiments. Animals were humanely euthanized when they displayed symptoms such as ruffled feathers and reluctance to move, not moving when prodded, respiratory distress, or injuries that were not related to experimental treatment. Euthanasia was actualized by exposure to carbon dioxide (CO_2_). Based on the age and body size, 1–10 animals were placed in the euthanasia chamber connected to a CO_2_ source. The CO_2_ flow was set at 10–30% displacement of chamber volume/minute. Birds were observed for respiratory arrest and the CO_2_ flow was maintained for at least one minute after the arrest was observed. The animals were checked for an absence of breathing and lack of heartbeat. If any respiration or heartbeat was detected, the animal was placed back into the chamber and additional CO_2_ was administered as described above. After death has been confirmed, an additional secondary physical euthanasia (cervical dislocation or removal of a vital organ) was performed before collection of tissues and carcass disposal.

### Experimental design

#### Design of study 1

The design of study 1 is summarized in Table 1. Briefly, fifty-four 1-day-old birds were split into three groups (LAIV, IIV, and unvaccinated) (n = 23 birds per group). Each bird in the LAIV group was intranasally vaccinated with 1×10^6^ EID_50_ doses of the vaccine virus diluted in phosphate-buffered saline (PBS) to a final volume of 200 μL. The IIV group received a mixture of IIV prepared from a stock of A/TK/OR/71 (TK/OR/71) (H7N3) (the parental virus of pc4-LAIV) with a titer of 2^9^ hemagglutinating units (HAUs) and Montanide ISA 70 adjuvant (Seppic, France) (IIV:Adjuvant = 3:7, V/V) via subcutaneous route. The infectious titer of the live TK/OR/71 stock used for IIV preparation was 3.8 × 10^8^ EID_50_ before inactivation. Betapropiolactone was used to inactivate the virus for IIV preparation as previously described [37]. The unvaccinated control group did not receive any treatment. Although mock-adjuvant group was not included in this study, data generated from our previous experiments suggests the ISA 70 adjuvant does not have non-specific antiviral effects since the levels of challenge virus shedding with or without adjuvant were within the same range [32, 38]. All birds were monitored for clinical symptoms and behavioral changes throughout the study. At 1 and 3 days post-vaccination (dpv), five birds from each group were euthanized to collect trachea and spleen samples for use in transcriptional analysis as described below. At 14 dpv, 10 birds were randomly selected from each group and serum and tear samples were collected to measure systemic and mucosal antibody responses, respectively. After serum and tear collection, all birds were intranasally challenged with 1×10^6^ EID_50_ doses of a heterologous virus, A/CK/NJ/150383-7/02 (CK/NJ/02) (H7N2), in a 200 μL volume. The CK/NJ/02 virus had been used in our previous studies [19, 32, 39] and shares 87.5% amino acid sequence similarity in the HA1 protein with wildtype TK/OR/71 (H7N3) virus and NS1-truncated mutants pc2 or pc4 [39]. At 3 and 5 days post-challenge (dpc), six or seven birds from each group were euthanized to harvest tracheal tissue for titration of the challenge virus titers through real time RT-PCR as previously described [40, 41].

**Table 1:** Design of study 1.

Groups*	Vaccine
1d LAIV	LAIV (variant pc4 of A/TK/OR/71 H7N3)
1d IIV	Inactivated A/TK/OR/71 H7N3
Unvaccinated negative control	No treatment

*Vaccination was at 1 day of age, Live vaccine, LAIV, was administered via intranasal route. Inactivated vaccine, IIV, was mixed with adjuvant and administered via subcutaneous route.

#### Design of study 2

Eight different vaccine regimens were tested in the second experiment as summarized in Table 2 (n = 8 per group). Three categories of vaccination regimens were investigated: unvaccinated, single dose (groups 1d LAIV, 1d IIV, 3w LAIV, and 3w IIV), and prime-boost (groups LAIV-LAIV, LAIV-IIV, and IIV-IIV). The unvaccinated control group did not receive any treatment. The single vaccination groups were vaccinated only once either at 1 day (1d LAIV and 1d IIV) or 3 weeks (3w LAIV and 3w IIV) of age. The prime-boost groups (LAIV-LAIV, LAIV-IIV, and IIV-IIV) received the priming and boost vaccinations at 1 day and 3 weeks of age, respectively. Vaccine administration was conducted as described for study 1 above. The birds were bled at 4 and 5 weeks of age to measure post-vaccination serum antibody titers. Tears were collected at 5 weeks of age to measure post-vaccination mucosal antibody responses. All birds were challenged with CK/NJ/02 virus as described above. The level of challenge virus replication was monitored through real time RT-PCR titration of tracheal swab samples collected at 2 and 4 dpc.

**Table 2:** Design of study 2.

Groups	Prime vaccination at 1 of age	Boost vaccination at 3 weeks of age
1d LAIV	LAIV	-
1d IIV	IIV	-
3w LAIV	-	LAIV
3w IIV	-	IIV
LAIV-LAIV	LAIV	LAIV
LAIV-IIV	LAIV	IIV
IIV-IIV	IIV	IIV
Unvaccinated negative control	-	-

The live vaccine, LAIV, derived from TK/OR/71 (H7N3) virus was administered through intranasal route, while inactivated vaccine, a mixture of inactivated TK/OR/71 (H7N3) virus and adjuvant was administered into subcutaneous area.

### Transcriptional analysis

Trachea and spleen samples were homogenized in Trizol reagent and total RNA was extracted as previously described [32, 42]. Messenger RNA (mRNA) was selectively converted into cDNA using RT-PCR with oligo dT primer and subjected to quantitative PCR using SYBR GREEN system (Quanta, Gaithersburg, MD, USA). The primer sequences used for amplification of 2’,5’-OAS, Mx, IFN- α, IFN-β, and IFN-γ genes were previously published [43]. The ΔΔCt method was used to determine differential gene regulation using GAPDH gene as the internal control. Gene expression levels were calculated as fold changes over the unvaccinated control group as previously described [32, 42].

### Serologic assays

For analysis of serum antibodies, chickens were bled via wing veins and the blood was incubated overnight at room temperature for serum separation to occur. The separated sera were heat inactivated and stored at -20°C until used for hemagglutination inhibition (HI) test. The HI test was conducted in accordance with the recommendation of World Organization for Animal Health (OIE) [44]. Briefly, 50 μl of heat inactivated serum was serially diluted and mixed with an equal volume of antigen preparation containing 8 HAUs of virus. The serum-antigen mixture was incubated for 30 min at room temperature to form antigen-antibody complexes. Then, 50 μl of 1% turkey erythrocyte suspension was added in each well. The HI titer was reciprocally determined as the end-point dilution showing complete inhibition of hemagglutination. Birds were considered seroconverted when the serum showed a HI activity in dilution of 2^1^ or higher.

To determine cross-reactivity of serum with homologous and heterologous antigens, cross HI test was conducted on live or beta-propiolactone-inactivated virus (TK/OR/71 (H7N3) and CK/NJ/02 (H7N2) with cross-matching antisera produced by vaccination with inactivated antigen or live virus infection. Based on the cross HI titer, the percent antigenic relatedness was calculated as previously described by Archetti and Horsfall [45].

### Mucosal antibody analysis

Mucosal antibody responses were measured using tear samples collected according to a previously described method with slight modification [46]. Briefly, chickens were comfortably held with their eyelids open and fine sodium chloride crystals (less than 0.01g) were sprinkled onto each eye. Once lachrymation was induced, tears were carefully harvested using a micropipette attached with a sterile tip and immediately placed in tubes. For consistency, and to prevent antibody dilution, only the first 50 μL of induced-tears was collected. The tear samples were stored at -20 °C until used for enzyme-linked immunosorbent assay (ELISA).

All tear samples were diluted by a factor of 100 before use. Three different ELISA kits were used to detect antibodies in tears. Influenza A nucleoprotein (NP)-specific antibodies were measured using a competitive ELISA kit (Influenza A NP antibody inhibitor ELISA; Virusys Corporation, Sykesville, MD, USA) according to instructions provided by the manufacturer. The NP Reduction Index (NPRI) value was calculated based on the following formula: NPRI = (1- [Absorbance value (Abs) of samples—mean Abs value of diluent control]/[mean Abs of unvaccinated control tear samples- mean Abs value of diluent control]). Influenza virus-specific IgG response was measured by indirect ELISA kit (IDEXX AI Ab test; IDEXX Laboratories, Westbrook, ME, USA) in accordance with manufacturer’s instructions. Influenza virus-specific IgA response was measured using the same kit described for IgG except that the secondary antibody was replaced with 10000 fold diluted HRP-labeled Anti-Chicken IgA (α chain specific) (Gallus Immunotech, Inc., Fergus, ON, Canada). The level of IgG or IgA was represented as raw OD values.

### Avidity index

The avidity index was measured as previously described [47] using end point ELISA. Briefly, 96 well microtiter plates (Nunc MaxiSorp^®^, Thermo Fisher Scientific, Rochester, NY, USA) were coated with purified whole inactivated TK/OR/71 H7N3 virus at 4°C overnight. The following day, the plates were warmed to room temperature for 2 hours. From this point onward, all manipulations were done at room temperature. The wells were blocked with 200 μl of 5% nonfat dry milk in PBS for 2 hours. After washing with PBS, two sets of serially diluted sera (100 μl/well) were added and incubated for 2 hours. After washing three times, one set of samples was treated with 4M urea (100 μl/well) for an hour while the other set was incubated with PBS containing 0.05% Tween 20. The plates were washed three times followed by addition of goat anti-chicken horse reddish peroxidase (HRP) labeled IgG (KPL, Gaithersburg, MD, USA) secondary antibodies to all wells and incubated for 2 hours. The plates were washed six times and the substrate, tetramethylbenzidine peroxidase (TMB; KPL) (50 μl/well), was added and incubated for 15 minutes. The reaction was stopped with 1 M phosphoric acid (50 μl/well) and the plates were read at 450 nm (i-Mark ELISA Reader, Bio-rad, Life Science Research, Hercules, CA, USA). The OD value of dilution right before the start of the linear phase of the titration curve (without urea treatment) was chosen for the avidity index. The avidity index was defined as “the OD with urea treatment / OD without urea x100”.

### Statistical analysis

Differences in virus and antibody titers among groups were determined through one-way ANOVA, followed by post-hoc Tukey test for group by group comparison (GraphPad Software version 6.07, San Diego, CA, USA). For the transcription analysis study, the Mann-Whitney U test (GraphPad Software version 6.07) was used to detect statistical differences in fold changes among groups [42].

## Results

### Study 1: Efficacy of live and inactivated vaccines in 1-day-old chickens

We previously demonstrated that upregulation of IFN-related genes by influenza vaccine correlates well with rapid induction of adaptive immune responses and enhancement of protective efficacy in 4-week-old chickens [32]. To determine and compare the efficacy of live and inactivated vaccines in younger birds, 1-day-old birds were vaccinated intranasally with LAIV or subcutaneously with IIV as shown in Table 1. One bird in the IIV group died at 1 day after vaccination due to unknown reasons. The remaining birds, in all three groups, did not show abnormal behavior, clinical signs, or mortality for the entire duration of the experiment.

#### Interferon (IFN) and IFN stimulated gene (ISG) responses

**Fig 1:**
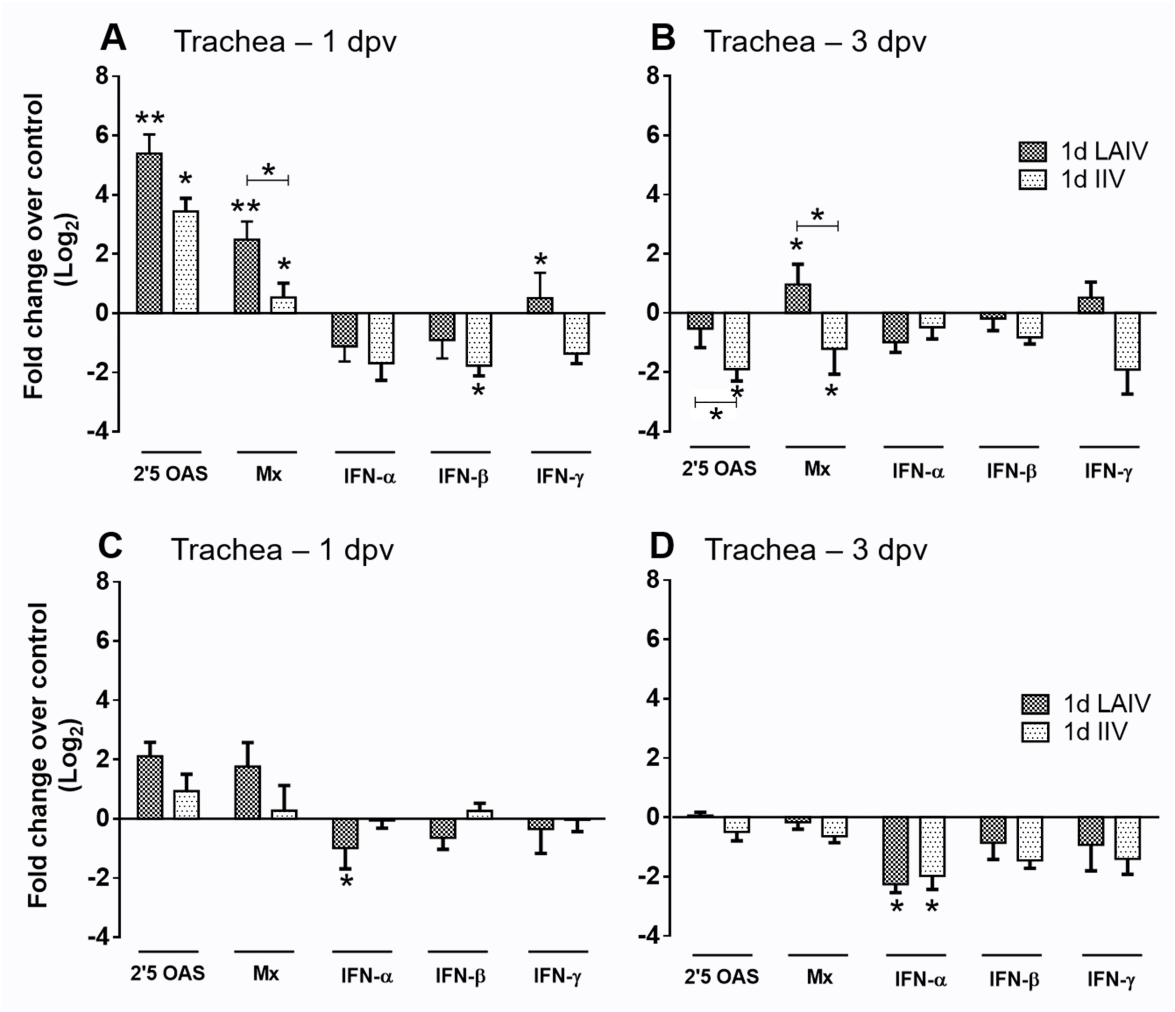
The ISG and IFN responses in chickens vaccinated with LAIV or IIV at 1 day of age. (A) Trachea tissue at 1 dpv. (B) Trachea tissue at 3 dpv. (C) Spleen tissue at 1 dpv. (D) Spleen tissue at 3 dpv. The bars represent Log_2_ fold change in transcription level of ISGs and IFN genes. Error bars represent mean±SD (n = 5 birds per group). Asterisks without horizontal bar indicate significant differences compared with unvaccinated group. Horizontal bar with asterisks indicates significant difference between LAIV and IIV (*p<0.*05*, **p<0.*01*). dpv = days post-vaccination.

The ability of LAIV and IIV to stimulate innate immune responses in 1-day-old chickens was assessed by quantification of mRNA transcription level of type I/II IFNs and ISGs. Fig 1 shows upregulation or downregulation of these genes as fold changes over the unvaccinated control group. The most consistent and apparent changes were observed in ISGs, 2’,5’-OAS and Mx genes. Both vaccines were able to induce a significant upregulation of 2’,5’-OAS in 1 dpv trachea and spleen tissues (Fig 1). However, there was no significant difference between the two vaccine groups despite having a lower magnitude of upregulation in the IIV group compared to the LAIV group. Transcription of Mx gene was also significantly increased by LAIV in trachea at both time points and in spleen at 1 dpv while induction of the Mx gene by IIV vaccination was observed only in 1 dpv tracheal samples (Fig 1). Birds that received LAIV had a significant decrease in IFN-α mRNA levels at both time points in spleen whereas those vaccinated with IIV had downregulated levels of IFN-β and IFN-γ transcription in trachea (at 1 dpv and 3 dpv, respectively) and IFN-α transcription in spleen at 3 dpv. Even though the level of 2’,5’-OAS gene transcription was consistently elevated in 1 dpv trachea and spleen samples by both kinds of vaccinations, the result from 3 dpv spleen samples showed that a significant downregulation of 2’,5’-OAS gene transcription was induced by IIV vaccination. Overall, ISG transcription was upregulated in both types of tissues at 1 dpv and reduced or downregulated at 3 dpv. There were subtle changes in IFN gene transcription but the general trend was downregulation in trachea at 1 dpv and in spleen at 3 dpv.

#### Pre-challenge antibody levels in serum and tears at 14 dpv

**Fig 2:**
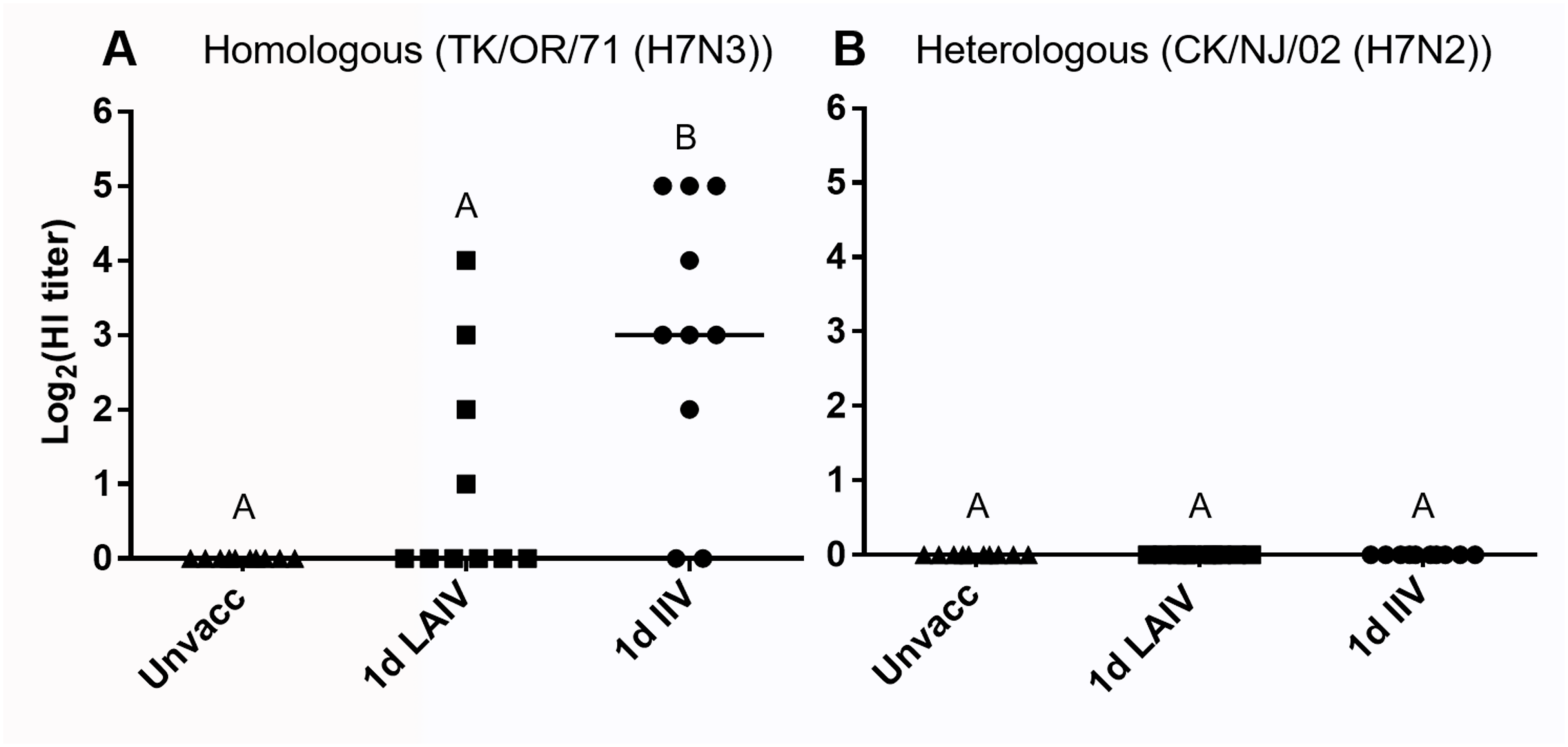
Serum HI antibody response in chickens vaccinated at 1 day of age. (A) Homologous (TK/OR/71, H7N3) HI titers. (B) Heterologous (CK/NJ/02(H7N2)) HI titers. Sera were collected from birds vaccinated at 1 day of age at 14 days post-vaccination. Individual and median HI titers are indicated with symbols and horizontal lines, respectively. Different letters inside the plot indicate statistical significance among groups (p<0.05).

**Fig 3:**
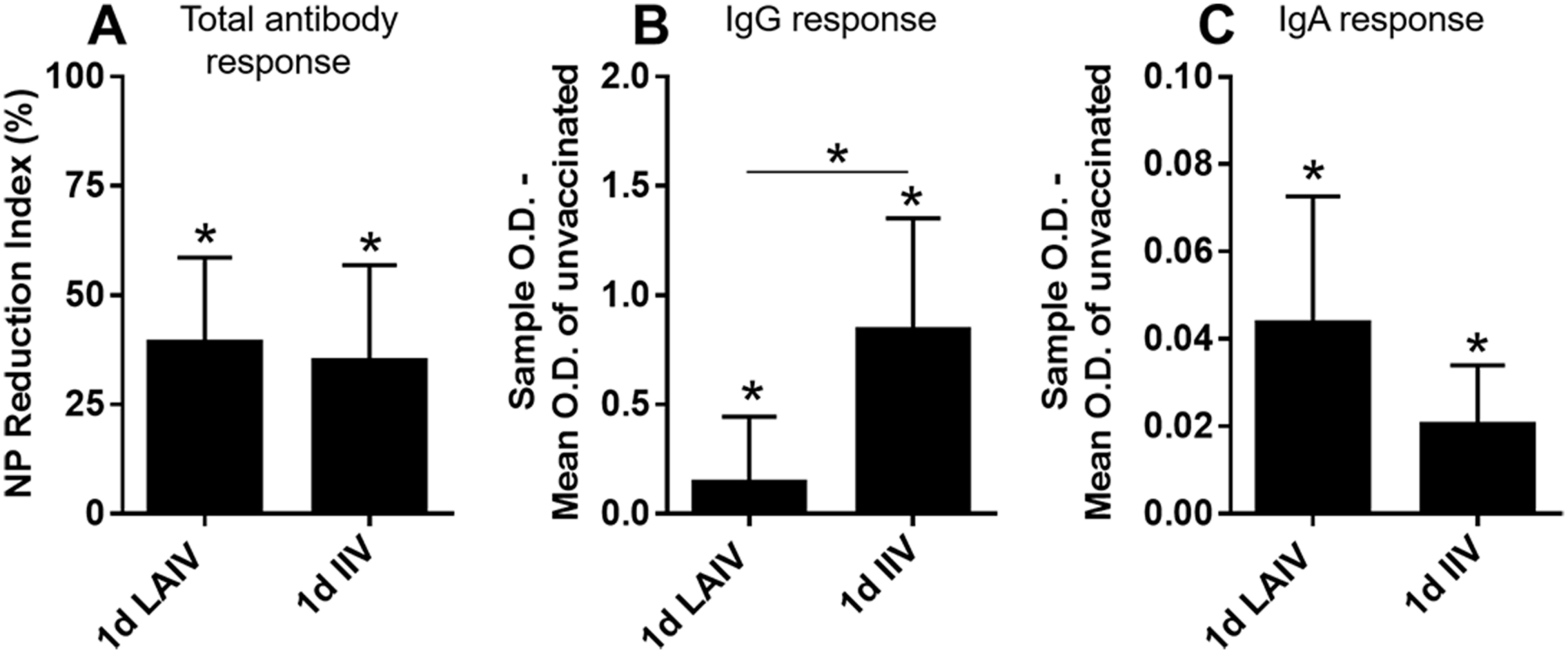
Mucosal antibody responses in tears of 2 week-old chickens. Birds were vaccinated at 1 day of age and tear samples collected at 14 days post-vaccination for antibody detection. (A) Total antibody against NP antigen. (B) Influenza virus-specific IgG. (C) Influenza virus-specific IgA. The NPRI and OD values shown on the y-axis were normalized to the mean value of the unvaccinated group. The asterisks indicate significant differences compared with unvaccinated group (*p<0.*05*, **p<0.*01*, and *** p<0.*001*).

Both kinds of vaccinations were poor at inducing serum antibody responses in 1-day-old chickens. Seroconversion to homologous virus (TK/OR/71 (H7N3)) was observed in eight out of ten birds in the IIV group and only four out of ten birds in the LAIV group. Heterologous (CK/NJ/02 (H7N2)) HI antibodies were not detected in any of the groups (Fig 2). Levels of antibodies in tear samples were measured using three kinds of ELISA kits which detect anti-influenza nucleoprotein (NP) antibody binding activity or the presence of influenza specific chicken immunoglobulins (IgG and IgA). The anti-NP ELISA kit is based on an inhibitory ELISA format, which detects any kind of antibodies that bind to the NP protein and presents the scale of antibody binding activity as NP Reduction Index (NPRI). As shown in Fig 3A, vaccinated groups had significantly higher NPRI values than the unvaccinated group. To further delineate the mucosal antibody responses induced by the two different forms of vaccine, the levels of influenza specific IgG and IgA responses were measured. Both LAIV and IIV vaccinations induced significant amounts of IgG antibodies and the level of induction was higher in the IIV group than the LAIV group (Fig 3B). While induction of IgA was observed in both vaccinated groups, only the LAIV group had significant IgA levels compared to the unvaccinated group (Fig 3C).

#### Heterologous protection efficacy

**Fig 4:**
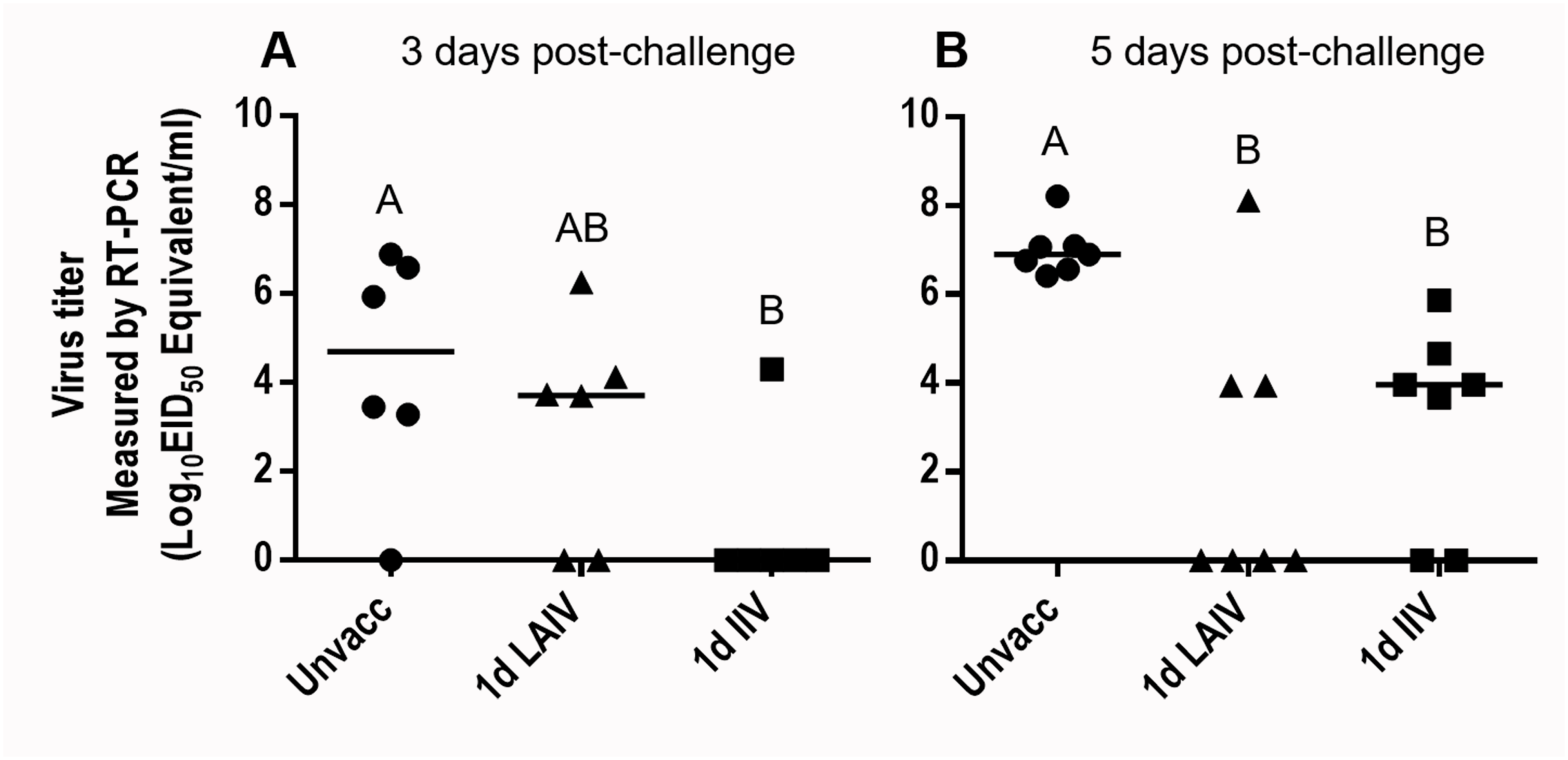
Replication level of heterologous challenge virus. Birds were vaccinated at 1 day of age and challenged with heterologous virus (CK/NJ/02 (H7N2)) at 2 weeks post-vaccination. Following challenge, full tracheas were collected and washed with 2 ml of PBS and the replication level of challenge virus was determined by qRT-PCR. Virus titers are expressed as median egg infectious doses per ml of tracheal wash supernatant. (A) 3 days post-challenge. (B) 5 days post-challenge. Different letters inside the plot indicate statistical significance among groups (p<0.05).

The above-described data (Figs 1, 2 and 3) suggested that, overall, LAIV and IIV stimulated the immune system of 1-day-old chickens via distinctive mechanisms. That is, LAIV induced higher levels of mucosal (tear) antibody (total antibody and IgA) responses (Fig 3A and 3C), whereas IIV was better at provoking systemic (serum) HI and mucosal IgG antibody responses. Interestingly, neither LAIV nor IIV could induce the high levels of serum antibodies that are usually associated with protective efficacy in older birds. To determine whether the observed immune responses can provide sufficient protection against heterologous wild type virus, the chickens were challenged with 1×10^6^ EID_50_ doses of a heterologous virus, CK/NJ/02 (H7N2), in a 200 μL volume at 2 weeks post vaccination (2 weeks of age) and the levels of challenge virus replication in trachea were compared among groups. CK/NJ/02 (H7N2) is heterologous to TK/OR/71 (H7N3) based on differences in HA1 sequences and low cross-reactivity of serum HI antibodies (Table 3) [39]. At 3 dpc, the median challenge virus titer of the unvaccinated control group was 10^4.69^ EID_50_ Equivalent/ml. A significant reduction in challenge virus replication was observed in the IIV group where virus was detected in only one out of six birds (Fig 4). The median challenge virus titer of the LAIV group was about 1 log lower than the unvaccinated group (10^3.71^ EID_50_ Equivalent/ml) but the difference was not statistically significant. As expected based on our previous study [32], the unvaccinated control group showed the highest level of challenge virus replication (10^6.89^ EID_50_ Equivalent/ml) at 5 dpc. Although virus replication was significantly reduced in vaccinated groups compared to the unvaccinated group, no virus was detected from more than half of the birds in the LAIV group (4/7) while more than half of birds in the IIV group (5/7) were virus positive with a median titer of 10^3.96^ EID_50_ Equivalent /ml (Fig 4).

**Table 3:** The effect of antisera production method on the percent antigenic relatedness value between TK/OR/71 (H7N3) and CK/NJ/02 (H7N2) antigens.

		H7N2 Antisera produced by	
		live virus infection	inactivated vaccination
Antisera produced by	Group 3w IIV	54.64	37.80
	Group 3w LAIV	49.42	34.18
	Group IIV-IIV	56.65	39.19
	Group LAIV-LAIV	63.09	43.64
	Group LAIV-IIV	67.49	46.69
	Group 1d IIV	55.86	38.36
	Group 1d LAIV	52.86	36.57

The cross HI test between vaccine strain (TK/OR/71, H7N3) and challenge strain (CK/NJ/02, H7N2) was conducted separately among antisera produced by live virus infection or inactivated vaccination. The cross HI test result was converted into percent antigenic relatedness as follows: Percent antigenic relatedness (R %) = 100 X $\sqrt{r1\;X\;r2}$; r1 = cross HI titer of H7N2 antisera to H7N3 antigen / HI titer of H7N3 antisera to H7N3 antigen; r2 = cross HI titer of H7N3 antisera to H7N2 antigen / HI titer of H7N2 antisera to H7N2 antigen [41].

### Study 2. Prime-boost regimens with LAIV and IIV

In study 1, although the types and levels of adaptive immune responses induced in young birds appeared to depend on the type of vaccine used, neither of the two vaccines could provide full protection at 5 dpc. Study 2 was designed to determine whether the beneficial effects from each vaccine can be exploited to produce a more efficacious vaccination regimen. Evidence obtained from our studies suggests that age is a critical determinant of the level of protection provided by heterologous influenza vaccines in chickens. For example, LAIV is more efficacious than IIV in 1-day-old birds (Study 1) whereas IIV is more efficacious in older birds [32]. In Study 2, our focus was on vaccination schedule consisting of priming with LAIV at 1 day of age and boosting with IIV at 3 weeks of age (Table 2, LAIV-IIV). This regimen was compared with other prime-boost regimens (IIV-IIV, LAIV-LAIV) or single (non-prime-boost) vaccinations (1d LAIV, 1d IIV, 3w LAIV, and 3w IIV) in terms of antibody response and heterologous protection efficacy (Table 2).

#### Serum HI antibody responses induced by different vaccination schedules

**Fig 5:**
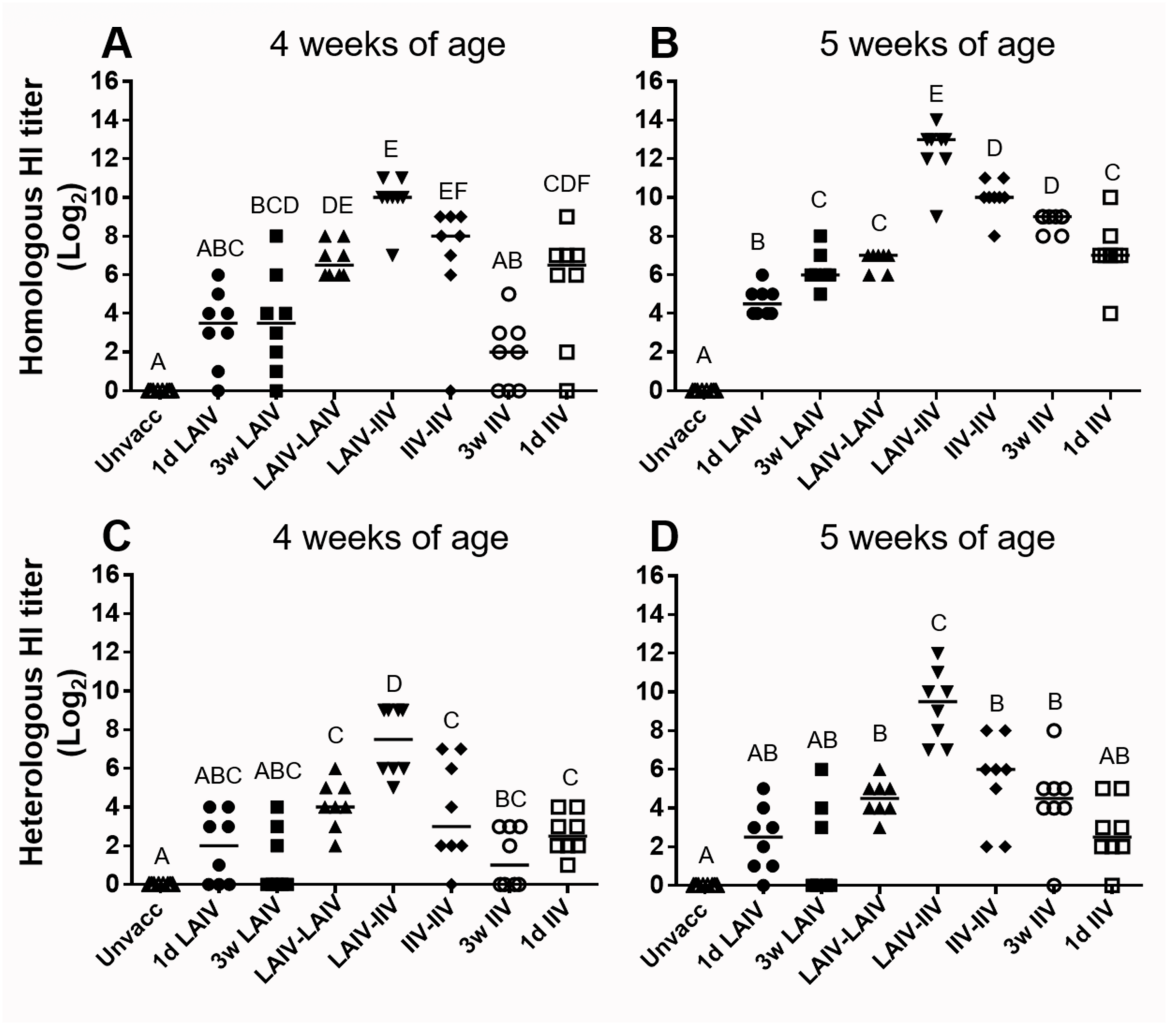
Serum antibody response to homologous (TK/OR/71 (H7N3)) and heterologous (CK/NJ/02 (H7N2)) strains at 4 and 5 weeks of age. (A) Homologous HI titers at 4 weeks of age. (B) Homologous HI titers at 5 weeks of age. (C) Heterologous HI titers at 4 weeks of age. (D) Heterologous HI titers at 5 weeks of age. Four weeks of age corresponded with 4 wpv for 1d IIV and 1d LAIV; 1 wpv for 3w LAIV and 3w IIV. Five weeks of age corresponded with 5 wpv for 1d IIV and 1d LAIV; 2 wpv for 3w LAIV and 3w IIV. The arrangement of groups highlights the synergy of LAIV priming and IIV boosting—LAIV-IIV is placed in the middle while other LAIV and IIV vaccinations are placed on the left and right, respectively. The individual and median HI titers are illustrated as symbols and horizontal lines, respectively. Different letters inside the plot indicate statistical significance among groups (p<0.05).

Fig 5 shows HI antibody responses at 4 and 5 weeks of age. Seroconversion of birds vaccinated at 1 day of age (1d IIV and 1d LAIV) was age-dependent and reached 100% for homologous viral antigen in 5-week-old chickens (Fig 5A and 5B). However, the concentration of heterologous HI serum antibodies remained either at a low level or at undetectable levels (Fig 5C and 5D). In birds vaccinated at 3 weeks of age, there was a clear difference between single vaccination regimens of live and inactivated vaccines (3w LAIV versus 3w IIV). All but one bird in the 3w LAIV group seroconverted to homologous viral antigen by 1-week post vaccination (wpv) and the median titer reached 2^4^ HI units (Fig 5A). At the same time point, three out of eight birds in the 3w IIV group failed to show homologous seroconversion and the median titer was 2^3^ HI units (Fig 5A). Homologous seroconversion was observed in all birds in both groups at 2 wpv but the trend of median HI titer was reversed: the 3w IIV group (2^9^ HI units) had a higher titer than the 3w LAIV group (2^6^ HI units) (Fig 5B). This result indicates that LAIV had the advantage of accelerating seroconversion whereas the IIV vaccination induced a higher level of antibody responses in chickens vaccinated at 3 weeks of age. As described above, although single vaccinations could induce homologous seroconversion in all birds at 5 weeks of age (5 wpv for 1d IIV and 1d LAIV; 2 wpv for 3w LAIV and 3w IIV), they did not induce 100% heterologous seroconversion. For the prime-boost regimen, the primary and boost vaccinations were administered at 1-day and 3 weeks of age, respectively (Table 2). At 1-week post-boost vaccination (wpb), both LAIV-LAIV and LAIV-IIV groups showed higher seroconversion rates (100%) for both homologous and heterologous strains compared to the IIV-IIV group (Fig 5A and 5C). The performance of the LAIV-IIV regimen was outstanding in terms of inducing the most rapid seroconversion and the highest HI serum antibody titers compared to the other groups (Fig 5).

#### Cross-reactivity of serum antibodies with homologous and heterologous viral antigens

**Fig 6:**
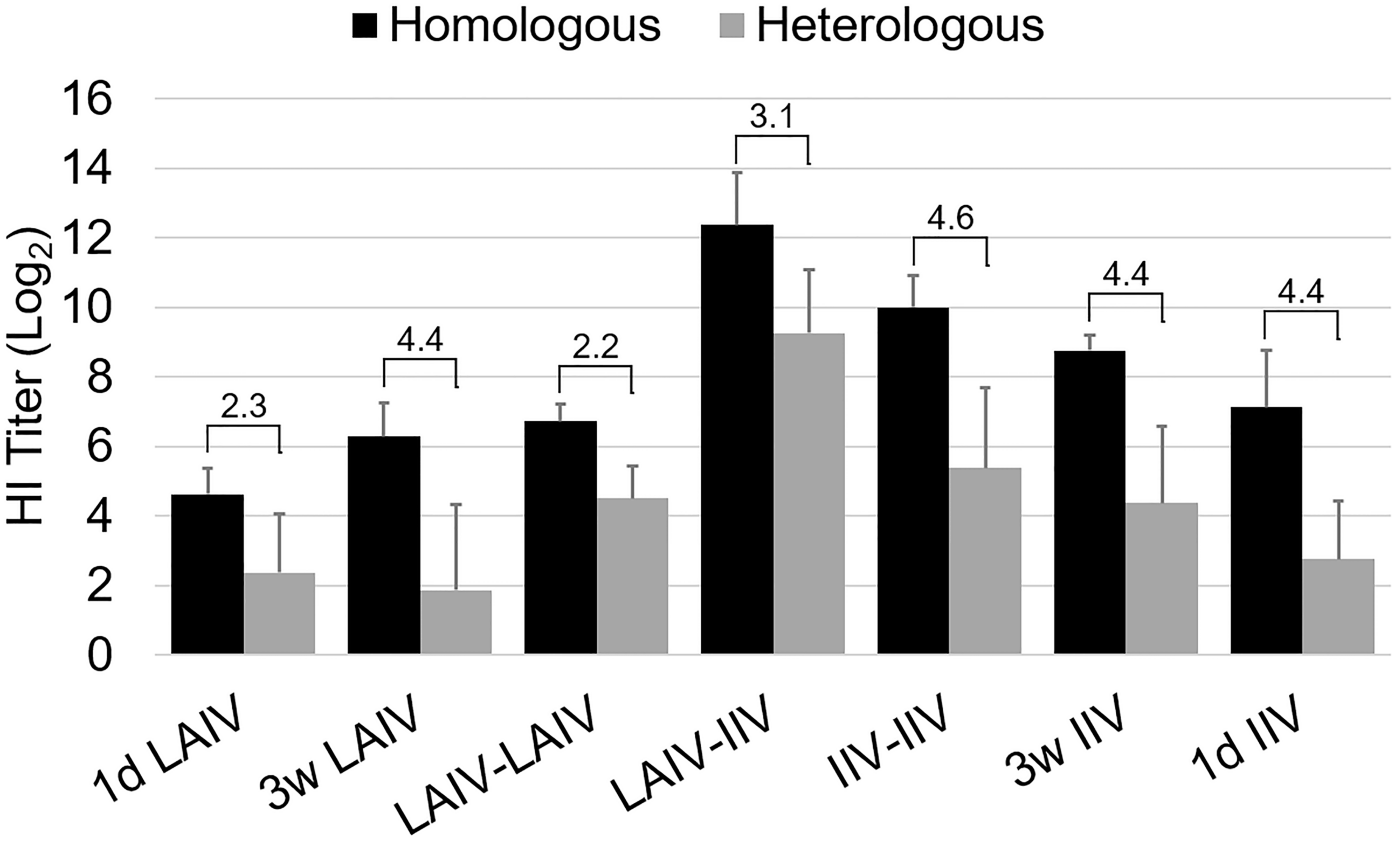
Differences in hemagglutination inhibition (HI) titers between homologous and heterologous strains. The bars and lines represent the average and standard deviation of Log_2_ HI titers at 5 weeks of age (5 wpv for 1d IIV and 1d LAIV; 2 wpv for 3w LAIV and 3w IIV). Numbers indicated above the bars and lines show average HI titer differences obtained by subtracting heterologous titers from homologous titers. Groups are arranged as described in Fig 5.

Both TK/OR/71 (the parental virus of LAIV-LAIV) and CK/NJ/02 viruses belong to the North American H7 lineage but they show low cross-reactivity to each other in cross HI test (Table 3) [39]. On average, HI titers against heterologous antigen were about 20-fold lower than homologous titers when hyper-immune sera prepared by IIV vaccination were used (Fig 5B and 5D). Interestingly, we observed in this study that the difference of HI titer between the vaccine and heterologous challenge strains was lower in sera from the groups primed with LAIV (LAIV-LAIV and LAIV-IIV) (Fig 6). For example, the HI titer difference between the two strains at 2 wpb was 2^2.2^ HI units in LAIV-LAIV group and 2^3.1^ HI units in LAIV-IIV group whereas HI titer differences in other groups were 2^4^ HI unit or higher, except 1d LAIV group (2^2.3^ HI units) (Fig 6). Since the HI titer difference for the IIV-IIV group was not different compared with IIV single vaccinations (1d IIV, 3w IIV), we reasoned that LAIV-LAIV priming was responsible for the broadened reactivity of the sera and thus results in smaller HI titer differences in LAIV-IIV and LAIV-LAIV groups. To further demonstrate the difference in reactivity of the sera among different vaccine groups, we conducted a cross-HI test and calculated the percent antigenic relatedness (R %) as previously described [45]. Table 3 summarizes the antigenic relatedness in a pairwise representation of the sera. We found consistently higher R values in antisera produced by live CK/NJ/02 (H7N2) virus infection than vaccination with inactivated vaccine (Table 3). R values were highest in the LAIV-IIV and LAIV-LAIV groups and low in IIV-IIV and single vaccination groups (Table 3).

#### Induction of mucosal influenza virus-specific antibody responses by different vaccine regimens

**Fig 7:**
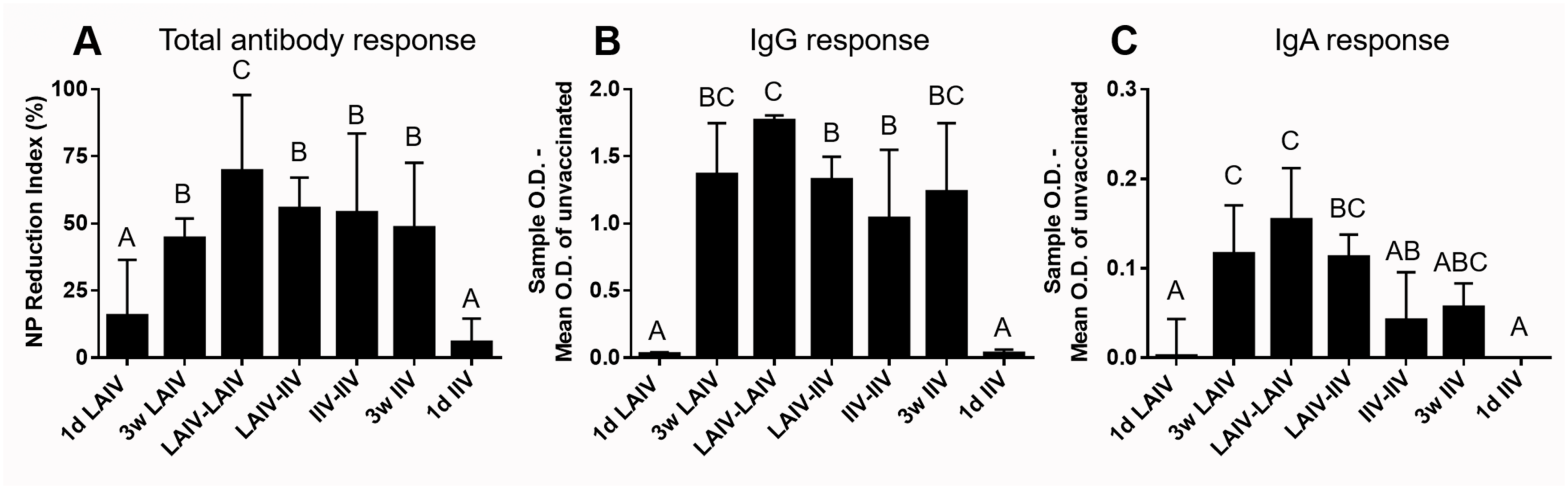
Mucosal antibody responses in tears of 5 weeks-old chickens. Birds were vaccinated at 1 day (1d) or 3 weeks (3w) of age. Tear samples were collected at 5 weeks of age (5 wpv for 1d IIV and 1d LAIV; 2 wpv for 3w LAIV and 3w IIV) to measure antibody levels. (A) Total antibody against NP antigen. (B) Influenza virus-specific IgG. (C) Influenza virus-specific IgA. The NPRI and OD values shown on the y-axis were normalized to the mean value of the unvaccinated group. Groups are arranged as described in Fig 5. Different letters inside the plot indicate significant differences between groups (p<0.05).

To compare the levels of mucosal antibody responses, we tested tear samples collected at 2 wpb vaccination with the three different ELISAs, as described above. Fig 7A and 7B show that all groups that received vaccination at 3 weeks of age had higher levels of total anti-NP and IgG antibodies compared with the unvaccinated control group. Fig 7C shows that vaccination with LAIV, as a priming or booster vaccine, resulted in induction of significantly higher levels of IgA responses compared with the groups vaccinated with IIV alone or the unvaccinated control group.

Overall, birds in the LAIV-LAIV group showed consistently high mucosal antibody responses across all three ELISA tests (Fig 7). The LAIV-IIV group also showed a good level of antibody response that was somewhat biased toward IgG response despite the fact that the IgA response observed in this group was not significantly different from the LAIV vaccinated groups (LAIV-LAIV and 3w LAIV). The single vaccination regimens administered at 3 weeks of age (3w IIV and 3w LAIV) showed a trend that was similar to the 1-day-old vaccination result: no differences in anti-NP or IgG antibodies (compare Fig 7A and 7B with Fig 3A and 3B) and higher IgA responses in LAIV groups compared with IIV groups (compare Fig 7C with Fig 3C).

#### Heterologous protection efficacy of prime-boost regimen

**Fig 8:**
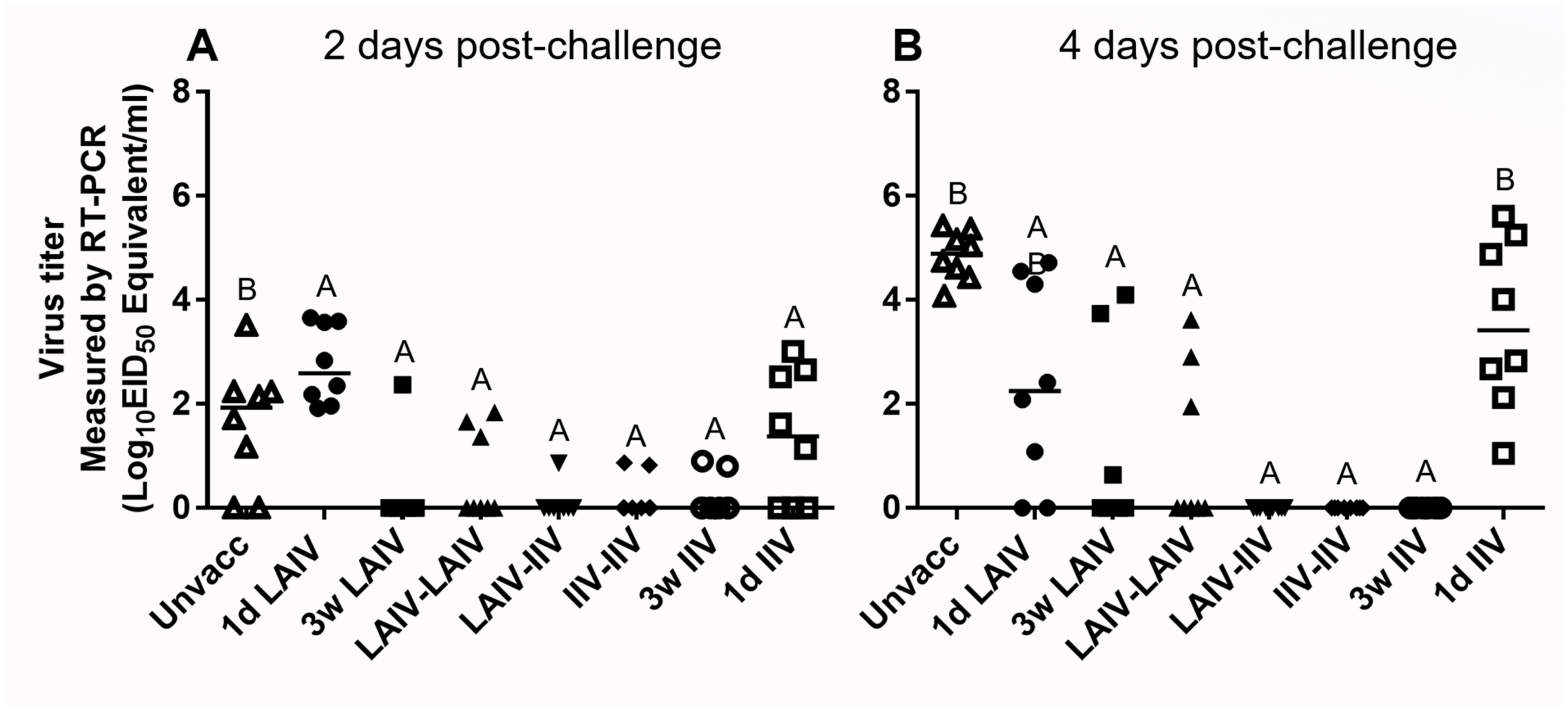
Reduction in heterologous challenge virus replication in vaccinated birds. At 5 weeks of age (5 wpv for 1d IIV and 1d LAIV; 2 wpv for 3w LAIV and 3w IIV), chickens were challenged with heterologous virus and tracheal swabs were taken at 2 and 4 days post challenge to determine the level of challenge virus replication. Each swab was eluted in 2 ml of PBS. Virus titers are expressed as median egg infectious doses per ml of tracheal swab eluate. (A) 2 days post-challenge. (B) 4 days post-challenge. Groups are arranged as described in Fig 5. Different letters inside the plot indicate significant differences between groups (p<0.05).

From the data presented above, it is clear that LAIV can increase the breadth of serum antibody reactivity and induce higher mucosal IgA responses than IIV. The prime-boost regimen using live and inactivated vaccines (LAIV-IIV) resulted in a synergistic effect that provided the highest serum antibody titer, an enhanced cross-reactivity of serum antibodies, and high levels of tear antibody responses. We further tested how those immune responses correlate with heterologous protective efficacy of each vaccine regimen. CK/NJ/02 virus was used as the heterologous challenge virus. The protective efficacy was assessed in terms of the ability of the vaccine regimen to reduce challenge virus replication in trachea compared to the unvaccinated control group. At 2 dpc, replication of the challenge virus was not high enough to enable comparison of the protective efficacies among vaccine regimens. At 4 dpc, the LAIV-IIV, IIV-IIV and 3w IIV regimens provided complete protection against heterologous challenge virus while the LAIV-LAIV regimen was partially, but significantly, protective (Fig 8). The reduction in challenge virus replication was not significant in the 1d LAIV and the 1d IIV groups, but still, two out of eight birds in the 1d LAIV group were able to prevent the replication of challenge virus (Fig 8). Interestingly, the 3w LAIV regimen was significantly protective (Fig 8) despite its inability to induce heterologous HI antibodies (Fig 5D) and having low R% values (Table 3).

#### Avidity of serum IgG antibodies

**Fig 9:**
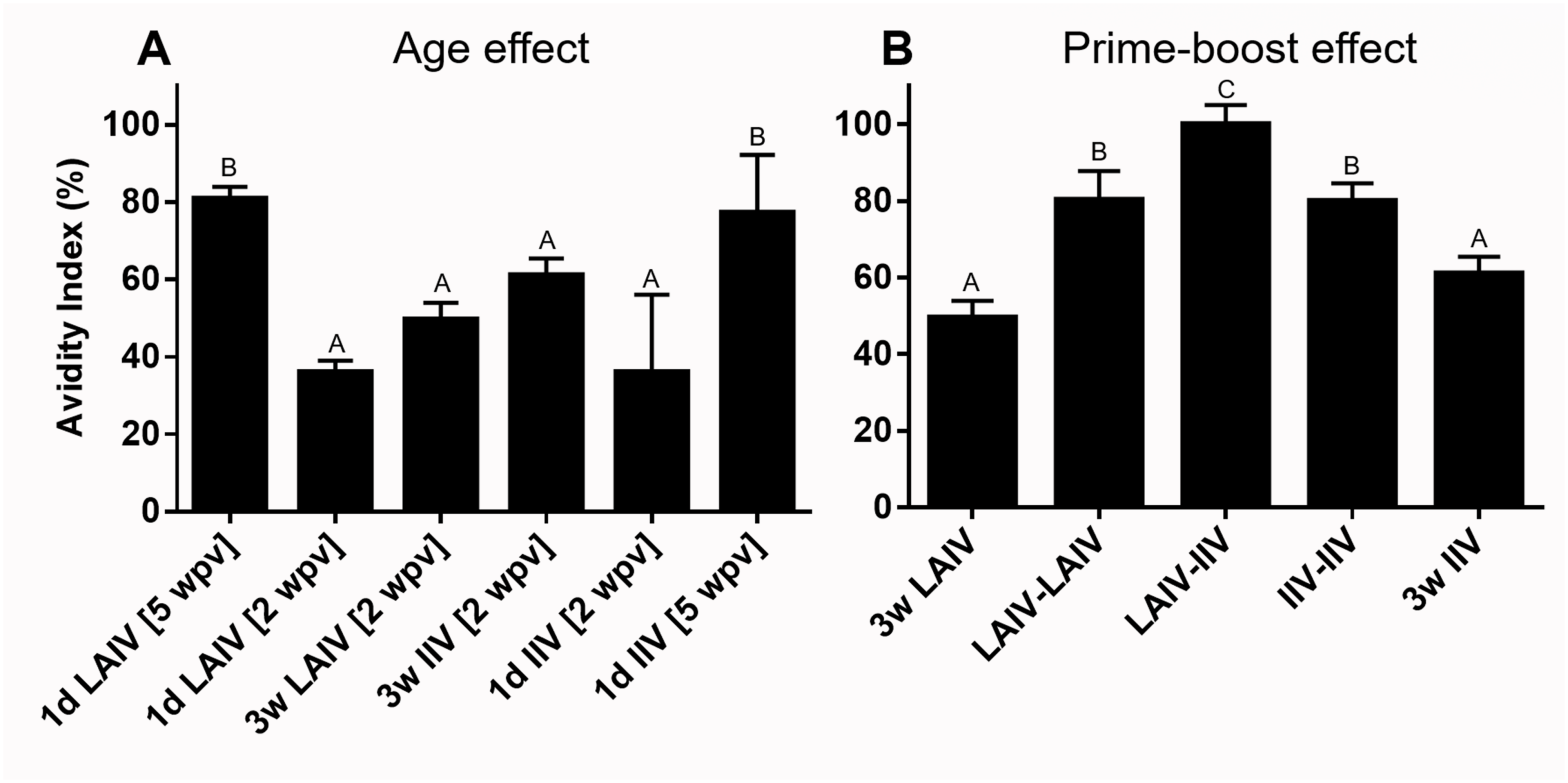
Effect of age and prime-boost vaccination on avidity index of serum HI antibodies. Serum samples collected at 2 or 5 weeks post vaccination at 1 day of age or 2 weeks post boost-vaccination (5 weeks of age) were tested for their avidity as previously described [43]. (A) Age effect. (B) Prime-boost effect. Groups are arranged as described in Fig 5. Different letters inside the plot indicate significant differences between groups (p<0.05).

After discovering that the vaccine type (LAIV or IIV) and vaccination regimen was influential on the quantity and heterologous cross-reactivity of serum HI antibodies (Figs 5 and 6, Table 3), we sought to determine the avidity of anti-influenza virus serum IgG antibodies. The avidity was lower in younger (2 week old) birds compared to older (5 week old) birds (Fig 9). Although the vaccine type did not have a significant effect on serum antibody avidity (compare 1d LAIV vs 1d IIV, 3w LAIV vs 3w IIV, and LAIV-LAIV vs IIV-IIV) and the avidity indices were generally 50% and above, the LAIV-IIV prime-boost regimen stood out by having a highest index of 100% (Fig 9).

## Discussion

Many vaccine effectiveness studies have confirmed that LAIV has advantages over IIV in terms of stimulating naïve and developing immune systems [26, 27, 48]. Live vaccines can efficiently trigger the innate immune system, via diverse pathogen-associated molecular patterns, and lead to regulation of several host genes including type I IFNs [49]. The type I IFN is a crucial component of the innate antiviral immune system which regulates several hundred ISGs and shapes the development of adaptive immune responses [50–53]. Two genes, 2’,5’-OAS and Mx, are common among the ISGs induced by type I IFNs in chickens [32, 35]. Upregulation of ISGs by LAIV correlates well with rapid induction of adaptive immune responses and enhancement of protective efficacy in 4-week-old chickens [32]. We were interested in determining whether LAIV can also be efficacious in younger immunologically naïve chickens. The evidence provided in the current study indicates that LAIV can elicit higher levels of innate responses, mucosal IgA antibodies, and heterologous protection in 1-day-old chickens compared to IIV. In addition, a prime-boost vaccination regimen utilizing both live and inactivated vaccines was shown to elicit a robust activation of systemic and mucosal adaptive immune responses and provide superior protection from heterologous challenge virus. This regimen could be a big advantage in young birds which have limited numbers of functional antigen presenting cells [54, 55]. In addition to the general advantages of live vaccine, our LAIV is highly immunogenic via the action of truncated NS1 protein as proved in our previous studies [19, 32].

We previously showed that the ability of NS1-truncated LAIV candidates to induce a rapid development of adaptive immune responses and protective efficacy in 4 week old chickens was strongly associated with upregulation of 2′,5′-OAS and Mx genes [32]. A similar pattern of ISG expression was observed in birds vaccinated at 1 day of age, especially in trachea at 1-day post-vaccination (Fig 1), which suggests that the performance of LAIV is not diminished in young immunologically immature chickens. Induction of 2′,5′ OAS and Mx gene upregulation in trachea was also seen in the IIV group, but the magnitude was lower compared to the LAIV group (Fig 1). This difference in ISG upregulation corresponds well with the differences in the level of mucosal IgA, serum antibody cross-reactivity, and protective efficacy induced by each vaccine. ISG upregulation by LAIV did not correspond with upregulation of IFN gene transcription in birds vaccinated at 1 day of age (Fig 1), which is the same observation previously made in birds vaccinated at 4 weeks of age [32]. We plan to investigate further on this discrepancy in our future studies. It should be noted that oral administration of recombinant chicken IFN-α was shown to mimic LAIV by inducing a rapid serum antibody response to IIV vaccination in our previous study [32].

A single administration of LAIV in 1-day old chickens was sufficient to induce a significant increase in influenza virus-specific IgA antibodies in tears but failed to stimulate high serum antibody titers. Previous studies have repeatedly described serum IgG and mucosal IgA responses as key features that distinguish live vaccines from parenterally administered inactivated vaccines [56–58]. The protection afforded by IIV vaccination primarily depends on the action of neutralizing serum antibodies and the serum HI antibody titer is a strong indicator of IIV efficacy [24]. Serum antibody response does not always correlate with protective efficacy of live vaccines. The underlying mechanism of serum antibody-independent viral clearance by live vaccines is postulated to be driven by mucosal IgA or CD8+ and CD4+ T cell responses [24, 59–61]. This serum antibody-independent viral clearance can be utilized to enhance vaccine efficacy in young chickens considering their poor antibody production ability [62, 63]. According to a study of live infectious bronchitis (IB) virus vaccination in chickens of different ages, birds vaccinated immediately after hatching tended to produce lower levels of serum IgG antibodies that had lower avidity indices compared to the birds vaccinated at 4-weeks of age [36]. Our data is in total agreement with the IB study in that the avidity of anti-influenza virus IgG is age-dependent (Fig 9A). Further, the avidity of antibodies induced by vaccination at 1-day of age continued to increase as the bird’s immune system matured (Fig 9A). Furthermore, the age-dependent IgG avidity was not significantly different between the intranasally delivered LAIV and parenterally administered IIV (Fig 9) implying that avidity may not be influenced by the mode of antigen delivery. Although we did not measure the avidity of mucosal IgA in the current study, the IB study did not find a significant effect of age on IgA avidity [36].

Another study of live IB vaccination in 1-day-old chickens also showed an inefficient antibody response and the protective efficacy of the vaccine mostly correlated with induction of high levels of CD4+, CD8+ and IgA bearing B cells [64]. Also, the vaccination of 1 day old chickens with adenovirus vectored H5 and H7 influenza vaccine could induce IgA response in lachrymal fluid and increased interleukin-6 expression without inducing detectable levels of serum antibodies [65]. Therefore, our current findings are consistent with previous reports in that LAIV enhances mucosal IgA response and provides viral clearance that is less dependent on serum antibody response in young chickens.

There were clear differences between the protective efficacy of LAIV and IIV in 1-day-old chickens. It was surprising to see that IIV could provide partial protection (Fig 4) without detectable levels of pre-challenge heterologous HI antibodies (Fig 2) considering that IIV provides protection mainly via neutralizing serum antibodies [66]. It remains to be investigated why IIV vaccination transiently provided an almost complete block of virus replication at 3 dpc (Fig 4). Reduction of challenge virus by LAIV vaccination was apparent at 3 dpc and statistically significant at 5 dpc when most of the birds were completely protected (Fig 4). Lau *et al* [67] also observed a similar protection trend with cold-adapted LAIVs in mice challenged with a heterologous virus at 28 dpv. A significant reduction in challenge virus titer in lung was observed at 4 dpc, but not at 2 dpc [67]. The differences between the protective efficacy of LAIV and IIV in 1-day-old chickens may reflect differences in immunologic mechanisms. For the protection of young, immunologically immature birds with a limited capacity to produce sufficient levels of serum antibodies, we believe that the LAIV could be the better option. Additional optimization, such as using mucosal adjuvant or modification of vaccination regimen and dosage, may further improve protective efficacy of LAIV in 1-day-old chickens.

Considering that both vaccines could protect most of the birds at 5 dpc (Fig 4), each vaccine provided protection by different mechanisms. We reasoned that these vaccines could supplement or even synergize each other if used in a prime-boost regimen. We have provided data to prove the advantage of this approach. When 1-day-old chickens were intranasally primed with LAIV and subcutaneously boosted with IIV three weeks later (LAIV-IIV vaccination), they showed a rapid, robust, and highly cross-reactive serum antibody response (Figs 5 and 6, Table 3) with high avidity (Fig 9) and a high level of mucosal IgA response (Fig 7). The LAIV-IIV regimen was remarkably synergistic in enhancing cross-reactivity with the heterologous antigen. For example, the mean heterologous HI titers induced by LAIV-IIV were at least 7 times higher than the mean HI titers of 1d LAIV and 3w IIV groups combined (Fig 5). This kind of synergy between live and inactivated vaccines was previously described in humans. Talaat *et al* [28] found that the frequency and level of antibody response to inactivated H5N1 vaccination was significantly higher in subjects who were previously primed with homologous H5N1 LAIV. After prime vaccination, there was no detectable serum antibody response but the immune system was apparently sensitized to rapidly respond to IIV vaccination and produce high titers of broadly cross-reactive serum HI antibodies [28]. More recently, Pitisuttithum *et al* [28] have confirmed induction of high serum HI antibody titers by IIV in LAIV-experienced individuals and went further to demonstrate that the antibody boosting effect correlated strongly with an increase in circulating follicular T-helper cells and plasma B cells. It appears that the long-lasting priming effect of LAIV has no species barrier.

Using the concept of antigenic relatedness based on cross-HI test [45, 68], we have demonstrated that priming with LAIV leads to induction of antibodies with enhanced cross-reactivity to heterologous virus independently of whether the boosting vaccine is live or inactivated (Table 3, compare LAIV-LAIV and LAIV-IIV with the other groups). In addition, the antigenic relatedness was higher with serum produced by live CK/NJ/02 (H7N2) virus infection relative to the hyper-immune serum produced by a matched IIV (Table 3, compare the columns). Therefore, the antibodies produced by live viral infection seem to have a stronger cross-reactivity to heterologous antigen. However, the HI antibodies from single LAIV vaccinations did not show an enhanced heterologous cross-reactivity (Table 3) and we attribute this to poor antibody induction (Fig 5). Enhancement of serum antibody cross-reactivity by LAIV vaccination or live virus infection has been reported in previous studies. Jang *et al* [69] reported that antibodies induced by the 2009 H1N1 pandemic influenza vaccine were cross-reactive with seasonal H1 and H5 strains. Hancock *et al* [70] found that persons 60 years or older had serum antibodies that cross-reacted with the 2009 H1N1 pandemic virus while sera from younger adults or children were not cross-reactive. The authors speculated that the cross-reactive antibodies found in elderly individuals were a result of priming by natural infection with H1N1 virus followed by vaccination with swine-origin A/NJ/76 H1N1 vaccine [70]. While the mechanism involved in induction of cross-reactive antibodies remain to be investigated, our findings in the current study and the observations made in the above-mentioned studies [69, 70] suggest the potential of LAIV priming as a strategy that can be exploited to develop a broadly effective influenza vaccination regimen.

All three groups that received IIV at 3 weeks of age (LAIV-IIV, IIV-IIV, and 3w IIV) developed high levels of serum HI antibodies (Fig 5) and fully blocked heterologous challenge virus replication at 4 dpc (Fig 8). Thus, the effects of the immunological advantages of the LAIV-IIV prime-boost regimen (Figs 5, 6 and 7, Table 3) could not be resolved at the level of challenge virus replication because full protection was observed in all 3 groups (Fig 3). However, the LAIV-IIV regimen may be the best option under field settings where circulating strains can be much more distantly related than the challenge virus used in this study.

## Conclusions

One of the perceived risks of vaccinating commercial poultry against influenza is the possibility of the vaccine protecting birds from disease without preventing replication and spread of the virus. We have demonstrated that a single dose of LAIV is able to induce stronger innate and mucosal IgA responses and protect young immunologically immature chickens better than a single dose of conventional IIV. Most importantly, priming with LAIV led to a synergistic serum antibody induction by IIV and enhancement of antibody cross-reactivity, thereby increasing the chance of protection from distantly related strains. Our prime-boost vaccine strategy requires further improvements to address possible limitations such as the cost effectiveness and safety issues. In addition, we are looking forward to evaluating the efficacy of the prime-boost regimen against different strains of HPAI viruses.

## Supporting information

S1 File Arrive guidelines checklist. (DOCX)
