# Supplementary material for: Efficacy and synergy of live-attenuated and inactivated influenza vaccines in young chickens
Source: PLoS One. 2018 Apr 6;13(4):e0195285. doi: 10.1371/journal.pone.0195285 (PMC5889186; doi:10.1371/journal.pone.0195285)
Supplement: S1 File — (DOCX) [file pone.0195285.s001.docx]

**The ARRIVE Guidelines**

**Animal Research: Reporting *In Vivo* Experiments**

|  | **Item** | | | **Recommendation** |  |
| --- | --- | --- | --- | --- | --- |
| **TITLE** | | **1** | **Efficacy and Synergy of Live-attenuated and Inactivated Influenza Vaccines in Young Chickens** | | |
| **ABSTRACT** | | **2** | **BACKGROUND AND PURPOSE:** Influenza virus causes a highly contagious disease in both humans and animals. Outbreaks of novel highly pathogenic avian influenza viruses have been reported in poultry species in the United States since 2014. These outbreaks have proven the limitations of biosecurity control programs, and new tools are needed to reinforce the current avian influenza control arsenal. Some enzootic countries have implemented inactivated vaccine (IV) in their control programs, but there are serious concerns that a long-term use of IV without eradication of heterologous strains may result in the selection of novel antigenically divergent strains. A broadly protective vaccine is needed, such as live-attenuated influenza vaccine (LAIV). We showed in our previous studies that pc4-LAIV (a variant that encodes a C-terminally truncated NS1 protein) can provide significant protection against heterologous challenge virus in chickens vaccinated at 2-4 weeks of age through upregulation of innate and adaptive immune responses. The current study was conducted to compare the performances of pc4-LAIV and IV in young chickens vaccinated at 1 day of age.  **EXPERIMENTAL APPROACH:**  **Transcriptional analysis**  Trachea and spleen samples were homogenized in Trizol reagent and total RNA was extracted as previously described [32, 42]. Messenger RNA (mRNA) was selectively converted into cDNA using RT-PCR with oligo dT primer and subjected to quantitative PCR using SYBR GREEN system (Quanta, Gaithersburg, MD, USA). The primer sequences used for amplification of 2’,5’-OAS, Mx, IFN- α, IFN-β, and IFN-γ genes were previously published [43]. The ΔΔCt method was used to determine differential gene regulation using GAPDH gene as the internal control. Gene expression levels were calculated as fold changes over the unvaccinated control group as previously described [32, 42].  **Serologic assays**  For analysis of serum antibodies, chickens were bled via wing veins and the blood was incubated overnight at room temperature for serum separation to occur. The separated sera were heat inactivated and stored at -20°C until used for hemagglutination inhibition (HI) test. The HI test was conducted in accordance with the recommendation of World Organization for Animal Health (OIE) [44]. Briefly, 50 µl of heat inactivated serum was serially diluted and mixed with an equal volume of antigen preparation containing 8 HAUs of virus. The serum-antigen mixture was incubated for 30 min at room temperature to form antigen-antibody complexes. Then, 50 µl of 1 % turkey erythrocyte suspension was added in each well. The HI titer was reciprocally determined as the end-point dilution showing complete inhibition of hemagglutination. Birds were considered seroconverted when the serum showed a HI activity in dilution of 2^1^ or higher.  To determine cross-reactivity of serum with homologous and heterologous antigens, cross HI test was conducted on live or beta-propiolactone-inactivated virus (TK/OR/71 (H7N3) and CK/NJ/02 (H7N2) with cross-matching antisera produced by vaccination with inactivated antigen or live virus infection. Based on the cross HI titer, the percent antigenic relatedness was calculated as previously described by Archetti and Horsfall [45].    **Mucosal antibody analysis**  Mucosal antibody responses were measured using tear samples collected according to a previously described method with slight modification [46]. Briefly, chickens were comfortably held with their eyelids open and fine sodium chloride crystals (less than 0.01g) were sprinkled onto each eye. Once lachrymation was induced, tears were carefully harvested using a micropipette attached with a sterile tip and immediately placed in tubes. For consistency, and to prevent antibody dilution, only the first 50 µL of induced-tears was collected. The tear samples were stored at -20 °C until used for enzyme-linked immunosorbent assay (ELISA).  All tear samples were diluted by a factor of 100 before use. Three different ELISA kits were used to detect antibodies in tears. Influenza A nucleoprotein (NP)-specific antibodies were measured using a competitive ELISA kit (Influenza A NP antibody inhibitor ELISA; Virusys Corporation, Sykesville, MD, USA) according to instructions provided by the manufacturer. The NP Reduction Index (NPRI) value was calculated based on the following formula: NPRI = (1- [Absorbance value (Abs) of samples – mean Abs value of diluent control]/[mean Abs of unvaccinated control tear samples- mean Abs value of diluent control]). Influenza virus-specific IgG response was measured by indirect ELISA kit (IDEXX AI Ab test; IDEXX Laboratories, Westbrook, ME, USA) in accordance with manufacturer’s instructions. Influenza virus-specific IgA response was measured using the same kit described for IgG except that the secondary antibody was replaced with 10000 fold diluted HRP-labeled Anti-Chicken IgA (α chain specific) (Gallus Immunotech, Inc., Fergus, ON, Canada). The level of IgG or IgA was represented as raw OD values.  **Avidity index**  The avidity index was measured as previously described [47] using end point ELISA. Briefly, 96 well microtiter plates (Nunc MaxiSorp®, Thermo Fisher Scientific, Rochester, NY, USA) were coated with purified whole inactivated TK/OR/71 H7N3 virus at 4°C overnight. The following day, the plates were warmed to room temperature for 2 hours. From this point onward, all manipulations were done at room temperature. The wells were blocked with 200 μl of 5% nonfat dry milk in PBS for 2 hours. After washing with PBS, two sets of serially diluted sera (100 μl/well) were added and incubated for 2 hours. After washing three times, one set of samples was treated with 4M urea (100 µl/well) for an hour while the other set was incubated with PBS containing 0.05% Tween 20. The plates were washed three times followed by addition of goat anti-chicken horse reddish peroxidase (HRP) labeled IgG (KPL, Gaithersburg, MD, USA) secondary antibodies to all wells and incubated for 2 hours. The plates were washed six times and the substrate, tetramethylbenzidine peroxidase (TMB; KPL) (50 μl/well), was added and incubated for 15 minutes. The reaction was stopped with 1 M phosphoric acid (50 μl/well) and the plates were read at 450 nm (i-Mark ELISA Reader, Bio-rad, Life Science Research, Hercules, CA, USA). The OD value of dilution right before the start of the linear phase of the titration curve (without urea treatment) was chosen for the avidity index. The avidity index was defined as ‘the OD with urea treatment / OD without urea x100’.    **Statistical analysis**  Differences in virus and antibody titers among groups were determined through one-way ANOVA, followed by post-hoc Tukey test for group by group comparison (GraphPad Software version 6.07, San Diego, CA, USA). For the transcription analysis study, the Mann-Whitney U test (GraphPad Software version 6.07) was used to detect statistical differences in fold changes among groups [42].  **MAJOR FINDINGS:**  We demonstrated that a single dose of pc4-LAIV was able to induce stronger local innate and mucosal IgA responses, protecting young, immunologically immature, chickens better than a single dose of IV. Most importantly, when 1-day-old chickens were intranasally primed with pc4-LAIV and subcutaneously boosted with IV three weeks later, they showed a rapid, robust, and highly cross-reactive serum antibody response and a high level of mucosal IgA antibody response. This vaccination regimen warrants further optimization to increase its range of protection. | |  |
| **INTRODUCTION** | |  |  | |  |
| - **Background** | | **3** | Avian influenza (AI) is a major zoonotic viral disease that causes significant adverse impacts on poultry production, the global trade, and public health [1-4]. Despite decades of research and control efforts, the incidences and severity of AI outbreaks have not been alleviated but rather increased [5, 6]. The strategies currently being employed to control AI are focused on prevention of virus introduction by maintaining strict biosecurity procedures [7-10]. However, the current biosecurity systems have repeatedly failed to protect poultry farms from introduction of novel strains that continue to cause major outbreaks [11, 12]. To overcome this challenge, some countries have incorporated vaccination with inactivated influenza vaccine (IIV) in their control programs [10, 13] . The United States has been using ‘stamping out’ as the primary control strategy, but the occurrence of recent highly pathogenic avian influenza (HPAI) outbreaks [13, 14] further proves the limitations of stamping out in combination with biosecurity control. For this reason, vaccination is being more seriously considered to be added to the country’s AI control arsenal. The IIV is currently in use in countries like Bangladesh, China, Egypt, Indonesia and Viet Nam where HPAI viruses are endemic [7]. Unfortunately, although IIV can provide good protection from homologous field strains [13], it is weak against heterologous strains that arise from random genetic mutations and not protective against heterosubtypic strains [9, 15, 16]. In addition to having a narrow range of protection, there are serious concerns that a long-term use of IIV without eradication of heterologous (mismatched) strains may result in the selection of antigenically divergent strains [17].  Live vaccines have numerous advantages over IIV that can be exploited further to develop new broadly protective vaccines and vaccination regimens. Since a live agent can mimic the natural infection, it can elicit a broad range of immune responses including humoral, cell-mediated and mucosal immunity [18-20]. Importantly, live vaccine can be directly administered on the mucosal surface by spray or through drinking water, which not only elicits local mucosal immunity but also significantly reduces the cost of mass administration [19, 21].  Currently, the only live influenza vaccines available for use in the poultry industry are live viral-vectored vaccines [10, 16]. Live viral-vectored vaccines can induce a broader range of protection compared to IIV, but they have several shortcomings including reduced efficacy due to preexisting immunity to the viral vector and the difficulty of expressing two or more influenza virus proteins in the same vector [16, 22, 23]. Live-attenuated influenza vaccine (LAIV) is an excellent alternative to the vectored vaccine or IIV since it contains all proteins that are naturally found in influenza virus particles [19, 24]. In humans, LAIV has been used for more than a decade and it has been reported to protect young individuals better than IIV [25-27]. Importantly, recent studies showed that LAIV can pre-sensitize the population and, subsequently, synergistically boost the efficacy of IIV [28, 29]. It should be noted that the use of LAIV in poultry requires strict safety standards due to concerns about the possibility that wild type strains may circulate in domestic poultry without apparent clinical symptoms, undergo genetic reassortment with the vaccine virus, and produce novel virulent strains [30, 31]. An ideal poultry LAIV should not be able to revert to wild type virus or undergo reassortment with field strains.  One of the promising candidates for poultry LAIV is pc4-LAIV, an influenza virus mutant that expresses a C-terminally truncated nonstructural protein 1 (NS1 protein) due to a large internal deletion (190 nt) in the NS gene segment, that caused a shift in the NS1 open reading frame and a premature stop codon [19]. Truncation of 137 amino acids from NS1 means that pc4-LAIV cannot easily revert to the wild type phenotype. In two independent vaccination studies, we demonstrated that pc4-LAIV is attenuated and does not transmit from chicken to chicken [19, 32]. Interestingly, even though pc4-LAIV did not replicate efficiently in young birds, it was able to induce robust adaptive immune responses and provide protection against heterologous virus infection [19, 32]. Available evidence suggests that the high level of immunogenicity of pc4-LAIV, despite its poor replication in chickens, is due to the deficiency of its NS1 in antagonizing the induction and signaling pathways of type I interferon (IFN) [32-34].  Influenza NS1 is known to block type I IFN response to create an environment that is favorable for virus replication and truncation of NS1 can lead to an increase in type I IFN production and attenuation of the virus in animals [20, 34]. We previously showed that infection with pc4-LAIV results in induction of high levels of type I IFN production in avian cells *in vitro* [33]. Although we were unable to detect IFN-α/β gene upregulation in vaccinated birds, there were strong correlations between interferon stimulated gene (ISG) upregulation, acceleration of serum antibody response, and induction of a high level of protection against heterologous virus in 4-week-old chickens [32]. In the same study, oral administration of recombinant chicken IFN-α was shown to mimic pc4-LAIV by inducing a rapid serum antibody response to IIV vaccination and an enhanced ISG response suggesting that pc4-LAIV efficacy is dependent on stimulation of the type I IFN system in the vaccinated host [32]. A similar effect was reported by Meng *et al* [35] in a study where oral treatment with recombinant chicken IFN-α resulted in induction of ISG transcription and inhibition of H9N2 influenza virus replication in 7 and 33-day-old chickens [35].  Live vaccines are more efficacious than inactivated vaccines in young, immunologically immature, chickens [36]. We have demonstrated that single vaccination with pc4-LAIV was highly immunogenic and protective in 2-4 weeks-old chickens [19, 32]. Based on our previous findings, the current study was conducted to compare the performances of pc4-LAIV (hereinafter referred to as LAIV) and IIV in young chickens vaccinated at 1 day of age. Although the performance of LAIV was better than IIV, and adaptive immune responses appeared to depend on the type of vaccine, neither of the vaccines could provide full protection against heterologous challenge virus. Subsequently, we designed and evaluated a vaccination regimen in which birds were primed with LAIV and boost-vaccinated with IIV at 1 day and 3 weeks of age, respectively. Not only did the prime-boost induce robust mucosal antibody responses, it also led to acceleration of seroconversion, synergistic increase of serum antibody titers, an increase in antibody cross-reactivity with heterologous antigen, and full protection from heterologous challenge virus. | |  |
|  | | **Relevance of Animal models** | Chickens are economically important species, and at the same time, they are the most susceptible species for avian influenza virus infection. The chicken is the best model for studying avian influenza vaccine for chickens. | |  |
| - **Objectives** | | **4** | 1. To compare the performances of live and inactivated influenza vaccines in young chickens vaccinated at 1 day of age. 2. Develop an optimal prime-boost vaccination regimen for young chickens using live and inactivated vaccines. | |  |
| **METHODS** | |  |  | |  |
| - **Ethical statement** | | **5** | **Ethical treatment of animals** All experimental animals were handled as previously described [32]. The animals were maintained, vaccinated, challenged and euthanized in accordance with protocol #2009AG0002 approved by The Ohio State University Institutional Animal Care and Use Committee (IACUC). This protocol complies with the U.S Animal Welfare Act, Guide for Care and Use of Laboratory Animals and Public Health Service Policy on Humane Care and Use of Laboratory Animals. The Ohio State University is accredited by the Association for the Assessment and Accreditation of Laboratory Animal Care International (AAALAC). White leghorn chickens were obtained from our institutional (Food Animal Health Research Program, Wooster, OH) specific pathogen free (SPF) flock. The chickens were housed in a BSL2 facility with forced air ventilation and adequate air exchanges to prevent ammonia build up. Air entering or leaving the facility is HEPA filtered. The birds were kept in large cages (2592 sq. inch) before infection and transferred to Model 934–1 isolators (900 sq. inch) (Federal Designs Inc., Comer, GA). The number of birds in each cage was calculated based on age and the space available after subtracting the space occupied by the feeder and the watering system. Room and isolator temperatures were maintained at 25±3°C. Birds had *ad libitum* access to feed and water. The wellbeing and health status of the animals was monitored twice daily throughout the experiments. Animals were humanely euthanized when they displayed symptoms such as ruffled feathers and reluctance to move, not moving when prodded, respiratory distress, or injuries that were not related to experimental treatment. Euthanasia was actualized by exposure to carbon dioxide (CO_2_). Based on the age and body size, 1–10 animals were placed in the euthanasia chamber connected to a CO_2_ source. The CO_2_ flow was set at 10–30% displacement of chamber volume/minute. Birds were observed for respiratory arrest and the CO_2_ flow was maintained for at least one minute after the arrest was observed. The animals were checked for an absence of breathing and lack of heartbeat. If any respiration or heartbeat was detected, the animal was placed back into the chamber and additional CO_2_ was administered as described above. After death has been confirmed, an additional secondary physical euthanasia (cervical dislocation or removal of a vital organ) was performed before collection of tissues and carcass disposal. | |  |
| - **Study design** | | **6** | **Design of study 1**  The design of study 1 is summarized in Table 1. Briefly, fifty-four 1-day-old birds were split into three groups (LAIV, IIV, and unvaccinated) (n = 23 birds per group). Each bird in the LAIV group was intranasally vaccinated with 1×10^6^ EID_50_ doses of the vaccine virus diluted in phosphate-buffered saline (PBS) to a final volume of 200 µL. The IIV group received a mixture of IIV prepared from a stock of A/TK/OR/71 (TK/OR/71) (H7N3) (the parental virus of pc4-LAIV) with a titer of 2^9^ hemagglutinating units (HAUs) and Montanide ISA 70 adjuvant (Seppic, France) (IIV:Adjuvant = 3:7, V/V) via subcutaneous route. The infectious titer of the live TK/OR/71 stock used for IIV preparation was 3.8 × 10^8^ EID_50_ before inactivation. Betapropiolactone was used to inactivate the virus for IIV preparation as previously described [37]. The unvaccinated control group did not receive any treatment. Although mock-adjuvant group was not included in this study, data generated from our previous experiments suggests the ISA 70 adjuvant does not have non-specific antiviral effects since the levels of challenge virus shedding with or without adjuvant were within the same range [32, 38]. All birds were monitored for clinical symptoms and behavioral changes throughout the study. At 1 and 3 days post-vaccination (dpv), five birds from each group were euthanized to collect trachea and spleen samples for use in transcriptional analysis as described below. At 14 dpv, 10 birds were randomly selected from each group and serum and tear samples were collected to measure systemic and mucosal antibody responses, respectively. After serum and tear collection, all birds were intranasally challenged with 1×10^6^ EID_50_ doses of a heterologous virus, A/CK/NJ/150383-7/02 (CK/NJ/02) (H7N2), in a 200 µL volume. The CK/NJ/02 virus had been used in our previous studies [19, 32, 39] and shares 87.5% amino acid sequence similarity in the HA1 protein with wildtype TK/OR/71 (H7N3) virus and NS1-truncated mutants pc2 or pc4 [39]. At 3 and 5 days post-challenge (dpc), six or seven birds from each group were euthanized to harvest tracheal tissue for titration of the challenge virus titers through real time RT-PCR as previously described [40, 41].  **Design of study 2**  Eight different vaccine regimens were tested in the second experiment as summarized in Table 2 (n=8 per group). Three categories of vaccination regimens were investigated: unvaccinated, single dose (groups 1d LAIV, 1d IIV, 3w LAIV, and 3w IIV), and prime-boost (groups LAIV-LAIV, LAIV-IIV, and IIV-IIV). The unvaccinated control group did not receive any treatment. The single vaccination groups were vaccinated only once either at 1 day (1d LAIV and 1d IIV) or 3 weeks (3w LAIV and 3w IIV) of age. The prime-boost groups (LAIV-LAIV, LAIV-IIV, and IIV-IIV) received the priming and boost vaccinations at 1 day and 3 weeks of age, respectively. Vaccine administration was conducted as described for study 1 above. The birds were bled at 4 and 5 weeks of age to measure post-vaccination serum antibody titers. Tears were collected at 5 weeks of age to measure post-vaccination mucosal antibody responses. All birds were challenged with CK/NJ/02 virus as described above. The level of challenge virus replication was monitored through real time RT-PCR titration of tracheal swab samples collected at 2 and 4 dpc. | |  |
| - **Experimental procedures** | | **7** | During the procedures, the birds were handled at least two personnel. One personnel gently held the bird into position while the other personnel administered agents, vaccine, etc.  **LAIV vaccination and challenge.**  One personnel gently held the bird into position while the other personnel opened the mouth and dropwise administered 0.2ml of virus via the intrachoanal-intranasal route using syringe with a blunted 22 gauge needle.  Vaccination with inactivated vaccine.  One personnel gently held the bird into position while the other personnel inoculated 0.2 ml of the vaccine subcutaneously (near the neck region) using syringe with a 22 gauge needle.  Blood Collection.  Blood was collected at intervals of not less than 1 week and immediately before euthanasia using syringes with 1 inch long, 22-26 gauge needles depending on the age of the animal. The birds were bled via wing (brachial) and jugular veins.   - The volume of blood drawn from each bird was in accordance with OSU IACUC blood collection policy and did not exceed 1% of the total body weight in a single draw or 1.5% of the total body weight over a 2 week period. - When more blood was required, the birds were euthanized, and then as much additional blood as possible was collected via cardiac puncture using an 18 gauge needle and syringe.   Swabbing.  Tracheal swabs were collected from the air passage (trachea) at the back of the bird’s mouth. To gain access to the opening of the trachea, the tongue was gently pulled forward to expose the trachea at the rear end of the tongue. The personnel taking the swab waited until the bird breathed and the cartilage protecting the trachea was open before inserting the swab and gently touching the sides and back of the trachea.  Cloacal swabs were collected by inserting the entire tip of the swab into the cloaca and swabbing with two to four circular motions while applying gentle pressure against the mucosal surfaces.  Euthanasia.   - The animals were placed in a container (tub). The lid (connected with a tube to a CO2 tank) was replaced and CO2 flow turned on. The rate of CO2 flow was set at 10-30% displacement of chamber volume/minute. - Birds were observed for respiratory arrest. - CO2 flow was maintained for at least one minute after respiratory arrest. - The animals were observed for an absence of breathing and lack or heart beat - If any respiration or heart beat was detected, the animal was placed back into tub and additional CO2 given as given as described above. - After confirmation of death, an additional secondary physical euthanasia (cervical dislocation, removal of vital organ etc) was performed before tissue collection and carcass disposal. | |  |
| - **Experimental animals** | | **8** | 1 day-old SPF chickens from FAHRP/OARDC flock.  All animals were tested and confirmed to be free of influenza virus by hemagglutination inhibition assay. | |  |
| - **Housing and husbandry** | | **9** | Chickens were housed in a BSL2 facility in a specific pathogen-free environment. The facility has forced air ventilation and adequate air exchanges to prevent ammonia build up. Air entering or leaving the facility is HEPA filtered. The birds were kept in large cages (2592 sq. inch) or in isolators (900 sq. inch). The number of birds in each cage was calculated based on age and the space available after subtracting the space occupied by the feeder and watering system. The room temperature was maintained at 25±3 °C. | |  |
| - **Sample size** | | **10** | Study 1: N=23 per group (5 euthanized at 1 day post vaccination, 5 euthanized at 3 day post vaccination, 6 euthanized at 3 days post challenge (dpc), 7 euthanized at 5 dpc) × 3 groups.  Study 2: N=8 per group (all birds included at all stages from vaccination to challenge) × 8 groups.  Based on work published by us and other major influenza research groups, these numbers are sufficient to provide accurate statistical power and interpretation of the data.  Selected References:  Lee CW, Senne DA, Suarez DL. Effect of vaccine use in the evolution of Mexican lineage H5N2 avian influenza virus. J Virol. 2004 Aug;78(15):8372-81.  Swayne DE, Lee CW, Spackman E. Inactivated North American and European H5N2 avian influenza virus vaccines protect chickens from Asian H5N1 high pathogenicity avian influenza virus. Avian Pathol. 2006 Apr;35(2):141-6.  Pillai SP, Pantin-Jackwood M, Suarez DL, Saif YM, Lee CW. Pathobiological characterization of low-pathogenicity H5 avian influenza viruses of diverse origins in chickens, ducks and turkeys. Arch Virol. 2010 Sep;155(9):1439-51.  Elaish M, Kang KI, Xia M, Ali A, Shany SA, Wang L, et al. Immunogenicity and protective efficacy of the norovirus P particle-M2e chimeric vaccine in chickens. Vaccine. 2015;33(38):4901-9. doi: 10.1016/j.vaccine.2015.07.049. PubMed PMID: 26232342.  Abdul-Careem, M. F., B. D. Hunter, A. J. Sarson, A. Mayameei, H. Zhou, and S. Sharif. 2006. Marek's disease virus-induced transient paralysis is associated with cytokine gene expression in the nervous system. Viral Immunol. 19:167-176. | |  |
| - **Allocating animals to experimental groups** | | **11** | 1-day-old chickens were randomly assigned to the experimental groups. | |  |
| - **Statistical methods** | | **12** | Differences in virus and antibody titers among groups were determined through one-way ANOVA, followed by post-hoc Tukey test for group by group comparison (GraphPad Software, San Diego, CA, USA). For the transcription analysis study, the Mann-Whitney U test (GraphPad Software) was used to detect statistical differences in fold changes among groups. | |  |
| **RESULTS** | |  |  | |  |
| - **Baseline data** | | **13** | The animals’ health status was monitored throughout the experiments by trained personnel. The LAIV candidates did not cause any obvious side effects. Further, the animals were confirmed to be specific pathogen-free through HI assay of anti-influenza virus antibodies in serum. | |  |
| - **Numbers analyzed** | | **14** | Data from 117 (Expt. 1 = 53, Expt. 2 = 64) chickens were included in the analyses. | |  |
| - **Outcomes and estimation** | | **15** | **Study 1: Efficacy of live and inactivated vaccines in 1-day-old chickens**  We previously demonstrated that upregulation of IFN-related genes by influenza vaccine correlates well with rapid induction of adaptive immune responses and enhancement of protective efficacy in 4-week-old chickens [32]. To determine and compare the efficacy of live and inactivated vaccines in younger birds, 1-day-old birds were vaccinated intranasally with LAIV or subcutaneously with IIV as shown in Table 1. One bird in the IIV group died at 1 day after vaccination due to unknown reasons. The remaining birds, in all three groups, did not show abnormal behavior, clinical signs, or mortality for the entire duration of the experiment.  **Interferon (IFN) and IFN stimulated gene (ISG) responses**  The ability of LAIV and IIV to stimulate innate immune responses in 1-day-old chickens was assessed by quantification of mRNA transcription level of type I/II IFNs and ISGs. Fig 1 shows upregulation or downregulation of these genes as fold changes over the unvaccinated control group. The most consistent and apparent changes were observed in ISGs, 2’,5’-OAS and Mx genes. Both vaccines were able to induce a significant upregulation of 2’,5’-OAS in 1 dpv trachea and spleen tissues (Fig 1). However, there was no significant difference between the two vaccine groups despite having a lower magnitude of upregulation in the IIV group compared to the LAIV group. Transcription of Mx gene was also significantly increased by LAIV in trachea at both time points and in spleen at 1 dpv while induction of the Mx gene by IIV vaccination was observed only in 1 dpv tracheal samples (Fig 1). Birds that received LAIV had a significant decrease in IFN-α mRNA levels at both time points in spleen whereas those vaccinated with IIV had downregulated levels of IFN-β and IFN-γ transcription in trachea (at 1 dpv and 3 dpv, respectively) and IFN-α transcription in spleen at 3 dpv. Even though the level of 2’,5’-OAS gene transcription was consistently elevated in 1 dpv trachea and spleen samples by both kinds of vaccinations, the result from 3 dpv spleen samples showed that a significant downregulation of 2’,5’-OAS gene transcription was induced by IIV vaccination. Overall, ISG transcription was upregulated in both types of tissues at 1 dpv and reduced or downregulated at 3 dpv. There were subtle changes in IFN gene transcription but the general trend was downregulation in trachea at 1 dpv and in spleen at 3 dpv.  **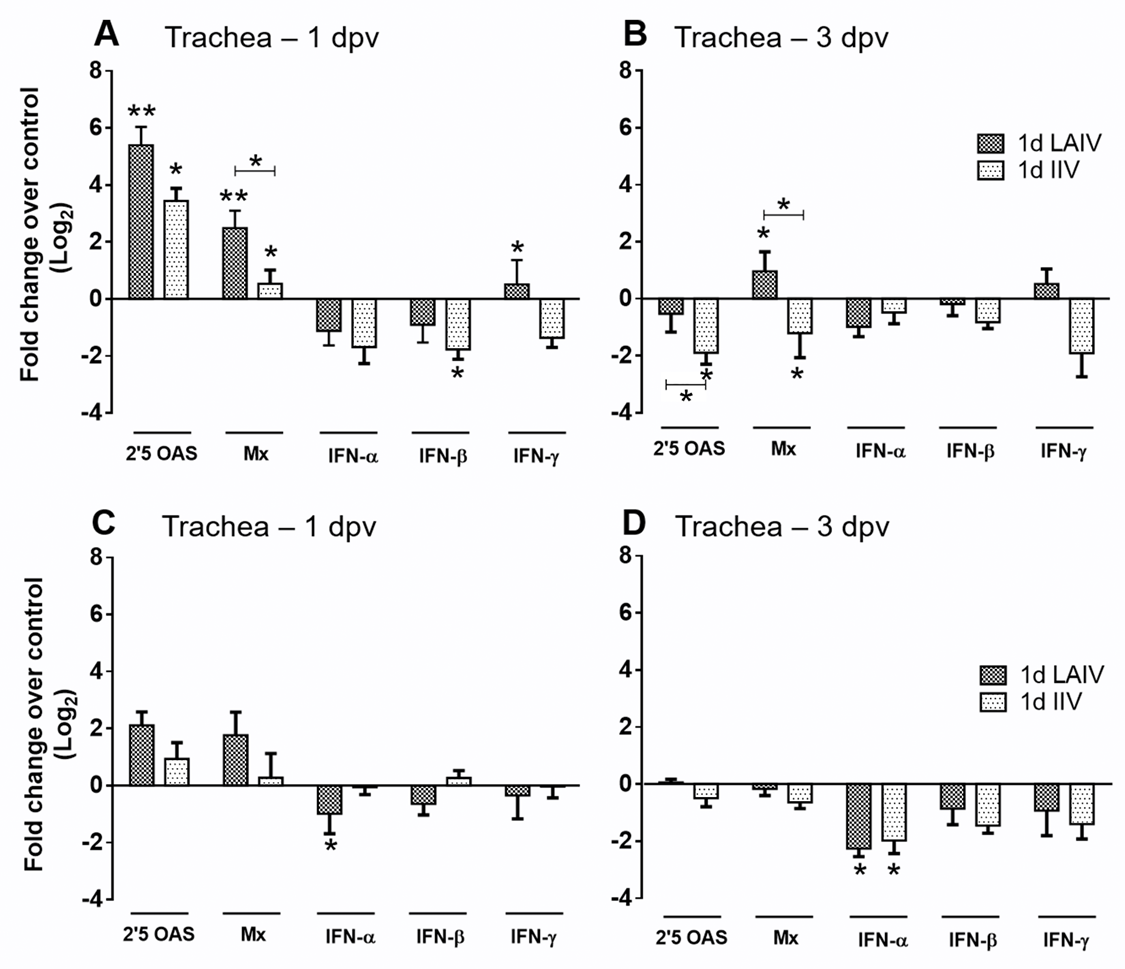**  **Fig 1. The ISG and IFN responses in chickens vaccinated with LAIV or IIV at 1 day of age.** (A) Trachea tissue at 1 dpv. (B) Trachea tissue at 3 dpv. (C) Spleen tissue at 1 dpv. (D) Spleen tissue at 3 dpv. The bars represent Log_2_ fold change in transcription level of ISGs and IFN genes. Error bars represent mean±SD (n=5 birds per group). Asterisks without horizontal bar indicate significant differences compared with unvaccinated group. Horizontal bar with asterisks indicates significant difference between LAIV and IIV (**p<0.05, **p<0.01*). dpv = days post-vaccination  **Pre-challenge antibody levels in serum and tears at 14 dpv**  Both kinds of vaccinations were poor at inducing serum antibody responses in 1-day-old chickens. Seroconversion to homologous virus (TK/OR/71 (H7N3)) was observed in eight out of ten birds in the IIV group and only four out of ten birds in the LAIV group. Heterologous (CK/NJ/02 (H7N2)) HI antibodies were not detected in any of the groups (Fig 2). Levels of antibodies in tear samples were measured using three kinds of ELISA kits which detect anti-influenza nucleoprotein (NP) antibody binding activity or the presence of influenza specific chicken immunoglobulins (IgG and IgA). The anti-NP ELISA kit is based on an inhibitory ELISA format, which detects any kind of antibodies that bind to the NP protein and presents the scale of antibody binding activity as NP Reduction Index (NPRI). As shown in Fig 3A, vaccinated groups had significantly higher NPRI values than the unvaccinated group. To further delineate the mucosal antibody responses induced by the two different forms of vaccine, the levels of influenza specific IgG and IgA responses were measured. Both LAIV and IIV vaccinations induced significant amounts of IgG antibodies and the level of induction was higher in the IIV group than the LAIV group (Fig 3B). While induction of IgA was observed in both vaccinated groups, only the LAIV group had significant IgA levels compared to the unvaccinated group (Fig 3C).  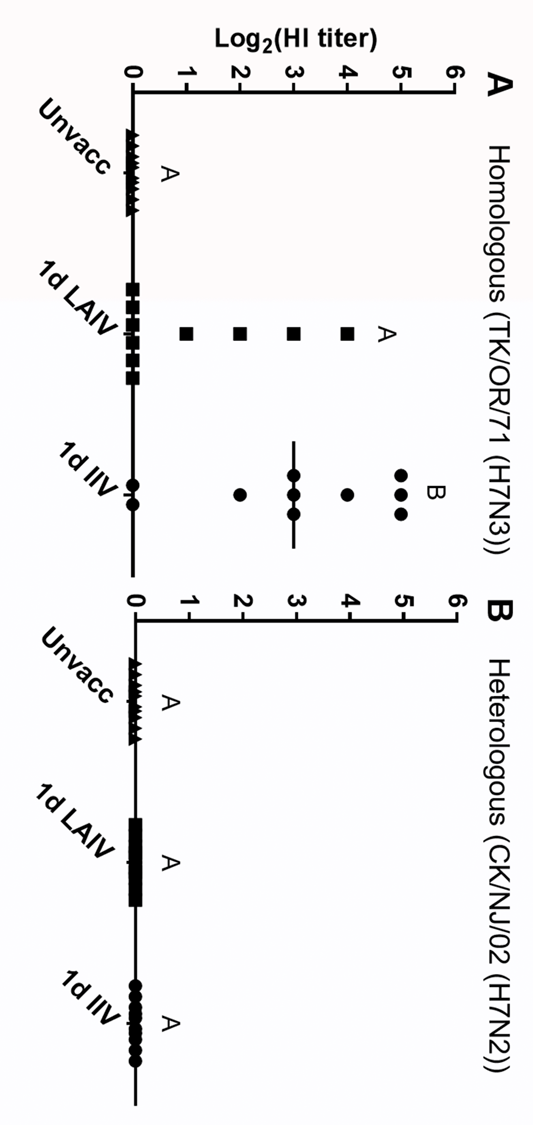  **Fig 2. Serum HI antibody response in chickens vaccinated at 1 day of age.** (A) Homologous (TK/OR/71, H7N3) HI titers. (B) Heterologous (CK/NJ/02(H7N2)) HI titers. Sera were collected from birds vaccinated at 1 day of age at 14 days post-vaccination. Individual and median HI titers are indicated with symbols and horizontal lines, respectively. Different letters inside the plot indicate statistical significance among groups (p<0.05).  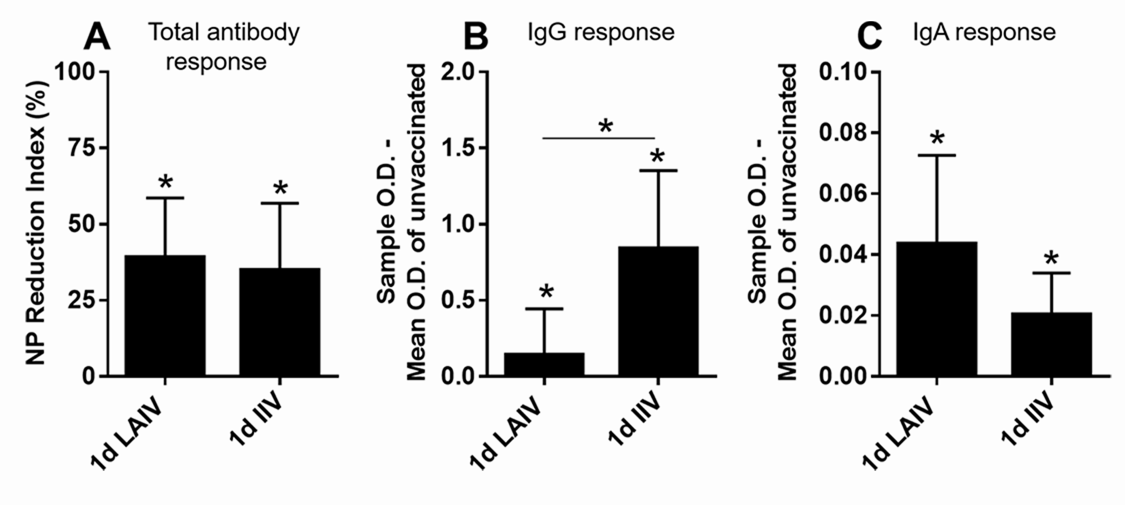  **Fig 3. Mucosal antibody responses in tears of 2 week-old chickens.** Birds were vaccinated at 1 day of age and tear samples collected at 14 days post-vaccination for antibody detection. (A) Total antibody against NP antigen. (B) Influenza virus-specific IgG. (C) Influenza virus-specific IgA. The NPRI and OD values shown on the y-axis were normalized to the mean value of the unvaccinated group. The asterisks indicate significant differences compared with unvaccinated group (**p<0.05, **p<0.01, and *** p<0.001*).  **Heterologous protection efficacy**  The above-described data (Figs 1, 2 and 3) suggested that, overall, LAIV and IIV stimulated the immune system of 1-day-old chickens via distinctive mechanisms. That is, LAIV induced higher levels of mucosal (tear) antibody (total antibody and IgA) responses (Figs 3A and 3C), whereas IIV was better at provoking systemic (serum) HI and mucosal IgG antibody responses. Interestingly, neither LAIV nor IIV could induce the high levels of serum antibodies that are usually associated with protective efficacy in older birds. To determine whether the observed immune responses can provide sufficient protection against heterologous wild type virus, the chickens were challenged with 1×10^6^ EID_50_ doses of a heterologous virus, CK/NJ/02 (H7N2), in a 200 µL volume at 2 weeks post vaccination (2 weeks of age) and the levels of challenge virus replication in trachea were compared among groups. CK/NJ/02 (H7N2) is heterologous to TK/OR/71 (H7N3) based on differences in HA1 sequences and low cross-reactivity of serum HI antibodies (Table 3) [39]. At 3 dpc, the median challenge virus titer of the unvaccinated control group was 10^4.69^ EID_50_ Equivalent/ml. A significant reduction in challenge virus replication was observed in the IIV group where virus was detected in only one out of six birds (Fig 4). The median challenge virus titer of the LAIV group was about 1 log lower than the unvaccinated group (10^3.71^ EID_50_ Equivalent/ml) but the difference was not statistically significant. As expected based on our previous study [32], the unvaccinated control group showed the highest level of challenge virus replication (10^6.89^ EID_50_ Equivalent/ml) at 5 dpc. Although virus replication was significantly reduced in vaccinated groups compared to the unvaccinated group, no virus was detected from more than half of the birds in the LAIV group (4/7) while more than half of birds in the IIV group (5/7) were virus positive with a median titer of 10^3.96^ EID_50_ Equivalent /ml (Fig 4).  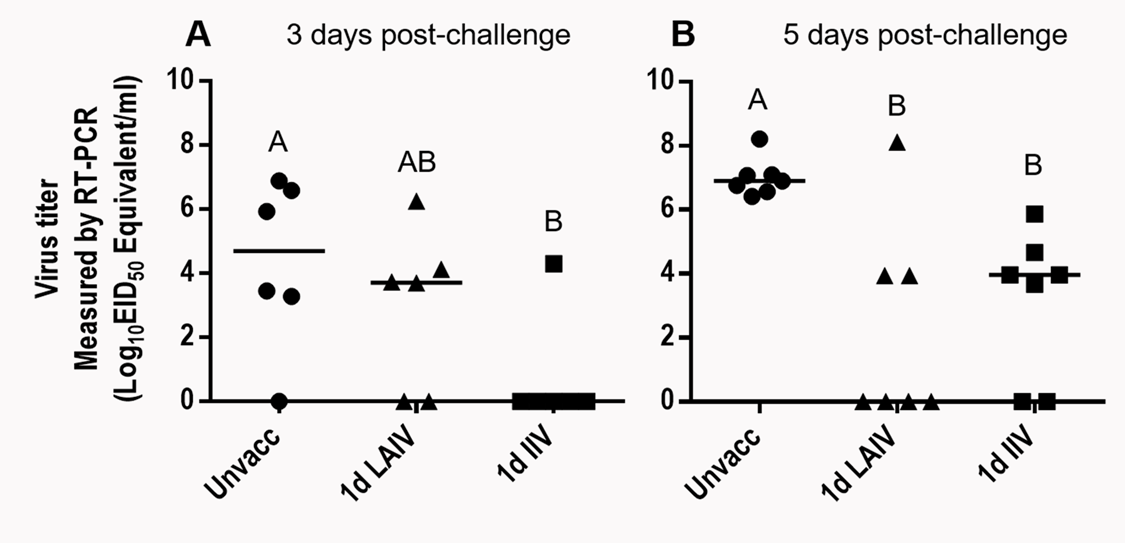  **Fig 4. Replication level of heterologous challenge virus.** Birds were vaccinated at 1 day of age and challenged with heterologous virus (CK/NJ/02 (H7N2)) at 2 weeks post-vaccination. Following challenge, full tracheas were collected and washed with 2 ml of PBS and the replication level of challenge virus was determined by qRT-PCR. Virus titers are expressed as median egg infectious doses per ml of tracheal wash supernatant. (A) 3 days post-challenge. (B) 5 days post-challenge. Different letters inside the plot indicate statistical significance among groups (p<0.05).  **Study 2. Prime-boost regimens with LAIV and IIV**  In study 1, although the types and levels of adaptive immune responses induced in young birds appeared to depend on the type of vaccine used, neither of the two vaccines could provide full protection at 5 dpc. Study 2 was designed to determine whether the beneficial effects from each vaccine can be exploited to produce a more efficacious vaccination regimen. Evidence obtained from our studies suggests that age is a critical determinant of the level of protection provided by heterologous influenza vaccines in chickens. For example, LAIV is more efficacious than IIV in 1-day-old birds (Study 1) whereas IIV is more efficacious in older birds [32]. In Study 2, our focus was on vaccination schedule consisting of priming with LAIV at 1 day of age and boosting with IIV at 3 weeks of age (Table 2, LAIV-IIV). This regimen was compared with other prime-boost regimens (IIV-IIV, LAIV-LAIV) or single (non-prime-boost) vaccinations (1d LAIV, 1d IIV, 3w LAIV, and 3w IIV) in terms of antibody response and heterologous protection efficacy (Table 2).  **Serum HI antibody responses induced by different vaccination schedules**  Fig 5 shows HI antibody responses at 4 and 5 weeks of age. Seroconversion of birds vaccinated at 1 day of age (1d IIV and 1d LAIV) was age-dependent and reached 100% for homologous viral antigen in 5-week-old chickens (Figs 5A and 5B). However, the concentration of heterologous HI serum antibodies remained either at a low level or at undetectable levels (Figs 5C and 5D). In birds vaccinated at 3 weeks of age, there was a clear difference between single vaccination regimens of live and inactivated vaccines (3w LAIV versus 3w IIV). All but one bird in the 3w LAIV group seroconverted to homologous viral antigen by 1-week post vaccination (wpv) and the median titer reached 2^4^ HI units (Fig 5A). At the same time point, three out of eight birds in the 3w IIV group failed to show homologous seroconversion and the median titer was 2^3^ HI units (Fig 5A). Homologous seroconversion was observed in all birds in both groups at 2 wpv but the trend of median HI titer was reversed: the 3w IIV group (2^9^ HI units) had a higher titer than the 3w LAIV group (2^6^ HI units) (Fig 5B). This result indicates that LAIV had the advantage of accelerating seroconversion whereas the IIV vaccination induced a higher level of antibody responses in chickens vaccinated at 3 weeks of age. As described above, although single vaccinations could induce homologous seroconversion in all birds at 5 weeks of age (5 wpv for 1d IIV and 1d LAIV; 2 wpv for 3w LAIV and 3w IIV), they did not induce 100% heterologous seroconversion. For the prime-boost regimen, the primary and boost vaccinations were administered at 1-day and 3 weeks of age, respectively (Table 2). At 1-week post-boost vaccination (wpb), both LAIV-LAIV and LAIV-IIV groups showed higher seroconversion rates (100%) for both homologous and heterologous strains compared to the IIV-IIV group (Fig 5A and C). The performance of the LAIV-IIV regimen was outstanding in terms of inducing the most rapid seroconversion and the highest HI serum antibody titers compared to the other groups (Fig 5).  **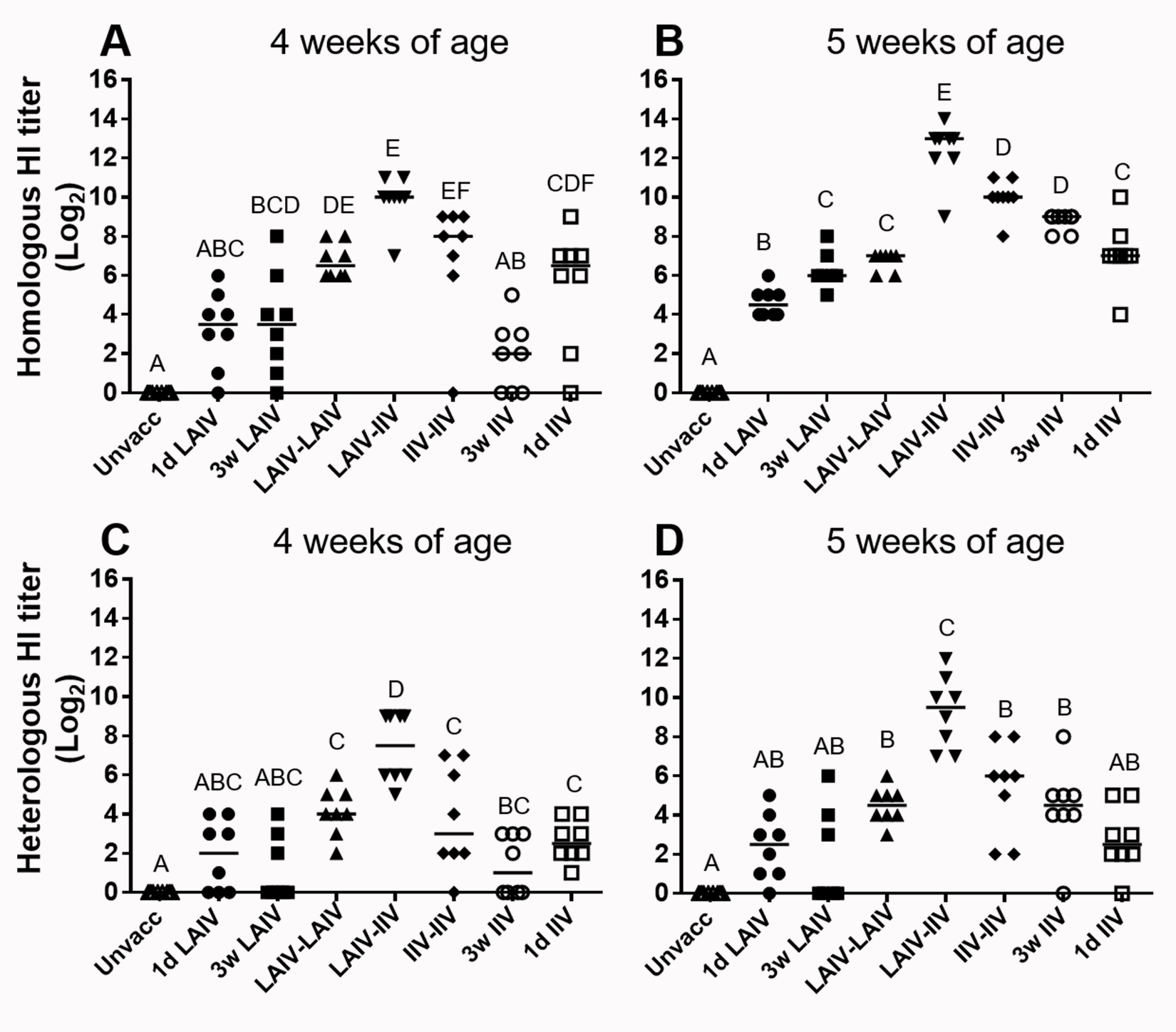**  **Fig 5. Serum antibody response to homologous (TK/OR/71 (H7N3)) and heterologous (CK/NJ/02 (H7N2)) strains at 4 and 5 weeks of age.** (A) Homologous HI titers at 4 weeks of age. (B) Homologous HI titers at 5 weeks of age. (C) Heterologous HI titers at 4 weeks of age. (B) Heterologous HI titers at 5 weeks of age. Four weeks of age corresponded with 4 wpv for 1d IIV and 1d LAIV; 1 wpv for 3w LAIV and 3w IIV. Five weeks of age corresponded with 5 wpv for 1d IIV and 1d LAIV; 2 wpv for 3w LAIV and 3w IIV. The arrangement of groups highlights the synergy of LAIV priming and IIV boosting – LAIV-IIV is placed in the middle while other LAIV and IIV vaccinations are placed on the left and right, respectively. The individual and median HI titers are illustrated as symbols and horizontal lines, respectively. Different letters inside the plot indicate statistical significance among groups (p<0.05).  C**ross-reactivity of serum antibodies with homologous and heterologous viral antigens**  Both TK/OR/71 (the parental virus of LAIV-LAIV) and CK/NJ/02 viruses belong to the North American H7 lineage but they show low cross-reactivity to each other in cross HI test (Table 3) [39]. On average, HI titers against heterologous antigen were about 20-fold lower than homologous titers when hyper-immune sera prepared by IIV vaccination were used (Figs 5B and 5D). Interestingly, we observed in this study that the difference of HI titer between the vaccine and heterologous challenge strains was lower in sera from the groups primed with LAIV (LAIV-LAIV and LAIV-IIV) (Fig 6). For example, the HI titer difference between the two strains at 2 wpb was 2^2.2^ HI units in LAIV-LAIV group and 2^3.1^ HI units in LAIV-IIV group whereas HI titer differences in other groups were 2^4^ HI unit or higher, except 1d LAIV group (2^2.3^ HI units) (Fig 6). Since the HI titer difference for the IIV-IIV group was not different compared with IIV single vaccinations (1d IIV, 3w IIV), we reasoned that LAIV-LAIV priming was responsible for the broadened reactivity of the sera and thus results in smaller HI titer differences in LAIV-IIV and LAIV-LAIV groups. To further demonstrate the difference in reactivity of the sera among different vaccine groups, we conducted a cross-HI test and calculated the percent antigenic relatedness (R %) as previously described [45]. Table 3 summarizes the antigenic relatedness in a pairwise representation of the sera. We found consistently higher R values in antisera produced by live CK/NJ/02 (H7N2) virus infection than vaccination with inactivated vaccine (Table 3). R values were highest in the LAIV-IIV and LAIV-LAIV groups and low in IIV-IIV and single vaccination groups (Table 3).  **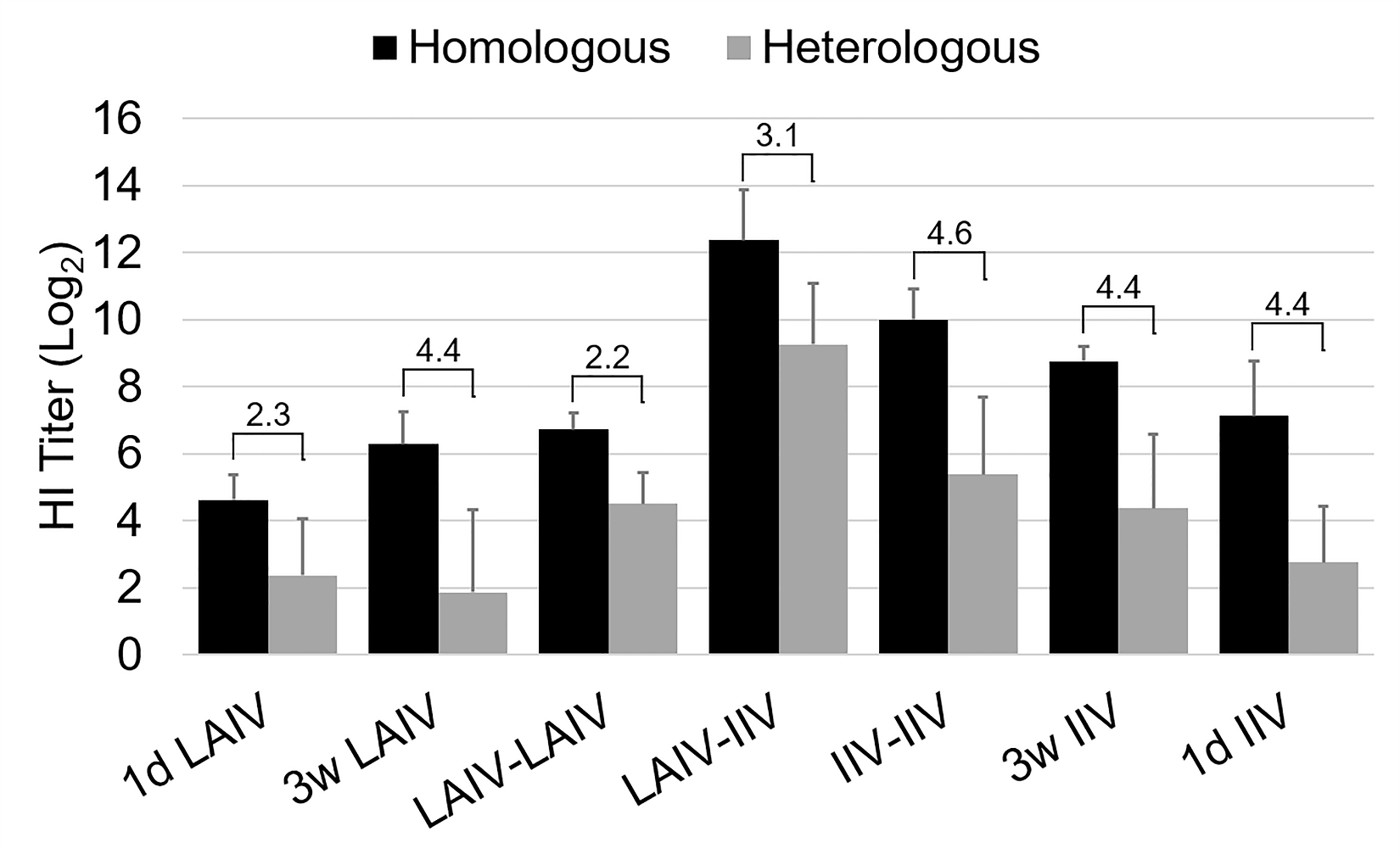**  **Fig 6. Differences in hemagglutination inhibition (HI) titers between homologous and heterologous strains.** The bars and lines represent the average and standard deviation of Log_2_ HI titers at 5 weeks of age (5 wpv for 1d IIV and 1d LAIV; 2 wpv for 3w LAIV and 3w IIV). Numbers indicated above the bars and lines show average HI titer differences obtained by subtracting heterologous titers from homologous titers. Groups are arranged as described in Fig 5.  **Table 3. The effect of antisera production method on the percent antigenic relatedness value between TK/OR/71 (H7N3) and CK/NJ/02 (H7N2) antigens**   \|  \| \| H7N2 Antisera produced by \| \| \| --- \| --- \| --- \| --- \| \| live virus infection \| inactivated vaccination \| \| Antisera produced by \| Group 3w IIV \| 54.64 \| 37.80 \| \| Group 3w LAIV \| 49.42 \| 34.18 \| \| Group IIV-IIV \| 56.65 \| 39.19 \| \| Group LAIV-LAIV \| 63.09 \| 43.64 \| \| Group LAIV-IIV \| 67.49 \| 46.69 \| \| Group 1d IIV \| 55.86 \| 38.36 \| \| Group 1d LAIV \| 52.86 \| 36.57 \|   The cross HI test between vaccine strain (TK/OR/71, H7N3) and challenge strain (CK/NJ/02 , H7N2) was conducted separately among antisera produced by live virus infection or inactivated vaccination. The cross HI test result was converted into percent antigenic relatedness as follows: Percent antigenic relatedness (R %) = 100 X $\sqrt{r1 X r2}$; r1 = cross HI titer of H7N2 antisera to H7N3 antigen / HI titer of H7N3 antisera to H7N3 antigen; r2 = cross HI titer of H7N3 antisera to H7N2 antigen / HI titer of H7N2 antisera to H7N2 antigen [41].  **Induction of mucosal influenza virus-specific antibody responses by different vaccine regimens**  To compare the levels of mucosal antibody responses, we tested tear samples collected at 2 wpb vaccination with the three different ELISAs, as described above. Figs 7A and 7B show that all groups that received vaccination at 3 weeks of age had higher levels of total anti-NP and IgG antibodies compared with the unvaccinated control group. Fig 7C shows that vaccination with LAIV, as a priming or booster vaccine, resulted in induction of significantly higher levels of IgA responses compared with the groups vaccinated with IIV alone or the unvaccinated control group.  **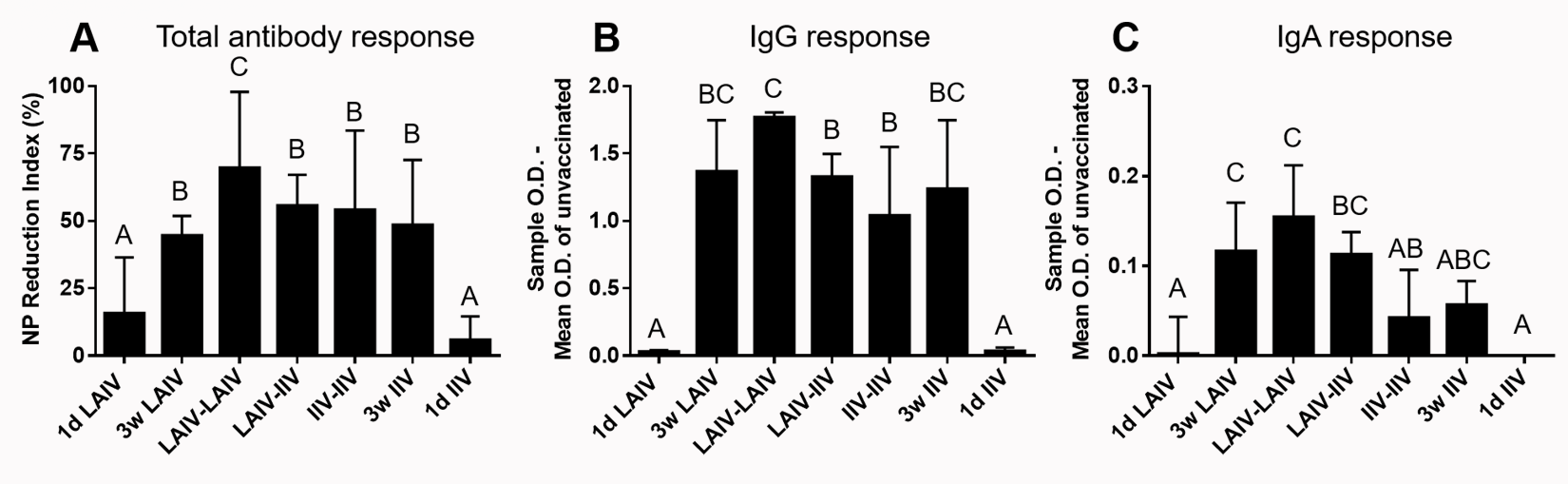**  **Fig 7. Mucosal antibody responses in tears of 5 weeks-old chickens.** Birds were vaccinated at 1 day (1d) or 3 weeks (3w) of age. Tear samples were collected at 5 weeks of age (5 wpv for 1d IIV and 1d LAIV; 2 wpv for 3w LAIV and 3w IIV) to measure antibody levels. Total antibody against NP antigen. (B) Influenza virus-specific IgG. (C) Influenza virus-specific IgA. The NPRI and OD values shown on the y-axis were normalized to the mean value of the unvaccinated group. Groups are arranged as described in Fig 5. Different letters inside the plot indicate significant differences between groups (p<0.05).  Overall, birds in the LAIV-LAIV group showed consistently high mucosal antibody responses across all three ELISA tests (Fig 7). The LAIV-IIV group also showed a good level of antibody response that was somewhat biased toward IgG response despite the fact that the IgA response observed in this group was not significantly different from the LAIV vaccinated groups (LAIV-LAIV and 3w LAIV). The single vaccination regimens administered at 3 weeks of age (3w IIV and 3w LAIV) showed a trend that was similar to the 1-day-old vaccination result: no differences in anti-NP or IgG antibodies (compare Fig 7A and B with Figs 3A and 3B) and higher IgA responses in LAIV groups compared with IIV groups (compare Figs 7C with 3C).  **Heterologous protection efficacy of prime-boost regimen**  From the data presented above, it is clear that LAIV can increase the breadth of serum antibody reactivity and induce higher mucosal IgA responses than IIV. The prime-boost regimen using live and inactivated vaccines (LAIV-IIV) resulted in a synergistic effect that provided the highest serum antibody titer, an enhanced cross-reactivity of serum antibodies, and high levels of tear antibody responses. We further tested how those immune responses correlate with heterologous protective efficacy of each vaccine regimen. CK/NJ/02 virus was used as the heterologous challenge virus. The protective efficacy was assessed in terms of the ability of the vaccine regimen to reduce challenge virus replication in trachea compared to the unvaccinated control group. At 2 dpc, replication of the challenge virus was not high enough to enable comparison of the protective efficacies among vaccine regimens. At 4 dpc, the LAIV-IIV, IIV-IIV and 3w IIV regimens provided complete protection against heterologous challenge virus while the LAIV-LAIV regimen was partially, but significantly, protective (Fig 8). The reduction in challenge virus replication was not significant in the 1d LAIV and the 1d IIV groups, but still, two out of eight birds in the 1d LAIV group were able to prevent the replication of challenge virus (Fig 8). Interestingly, the 3w LAIV regimen was significantly protective (Fig 8) despite its inability to induce heterologous HI antibodies (Fig 5D) and having low R% values (Table 3).  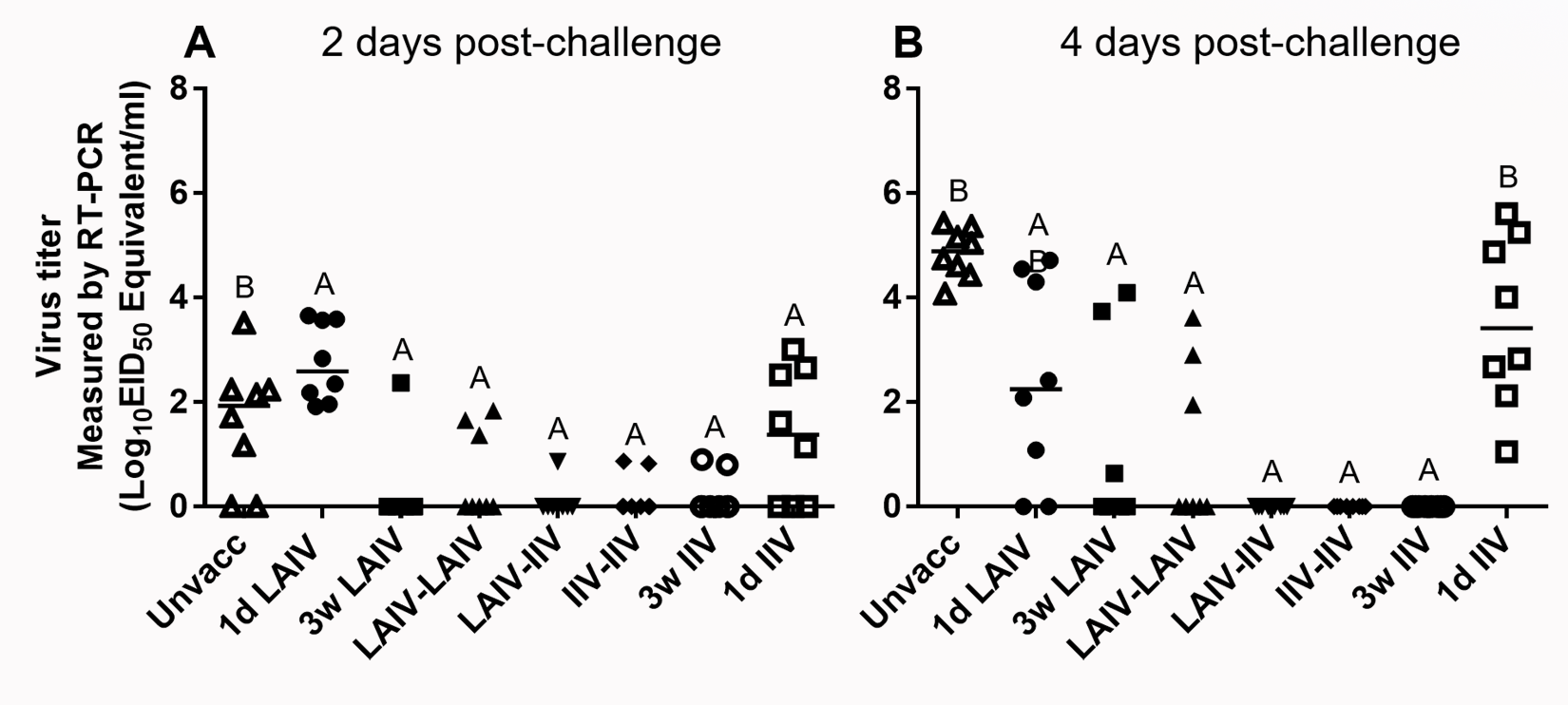  **Fig 8. Reduction in heterologous challenge virus replication in vaccinated birds.** At 5 weeks of age (5 wpv for 1d IIV and 1d LAIV; 2 wpv for 3w LAIV and 3w IIV), chickens were challenged with heterologous virus and tracheal swabs were taken at 2 and 4 days post challenge to determine the level of challenge virus replication. Each swab was eluted in 2 ml of PBS. Virus titers are expressed as median egg infectious doses per ml of tracheal swab eluate. (A) 2 days post-challenge. (B) 4 days post-challenge. Groups are arranged as described in Fig 5. Different letters inside the plot indicate significant differences between groups (p<0.05).  **Avidity of serum IgG antibodies**  After discovering that the vaccine type (LAIV or IIV) and vaccination regimen was influential on the quantity and heterologous cross-reactivity of serum HI antibodies (Figs 5 and 6, Table 3), we sought to determine the avidity of anti-influenza virus serum IgG antibodies. The avidity was lower in younger (2 week old) birds compared to older (5 week old) birds (Fig 9). Although the vaccine type did not have a significant effect on serum antibody avidity (compare 1d LAIV vs 1d IIV, 3w LAIV vs 3w IIV, and LAIV-LAIV vs IIV-IIV) and the avidity indices were generally 50% and above, the LAIV-IIV prime-boost regimen stood out by having a highest index of 100% (Fig 9).  **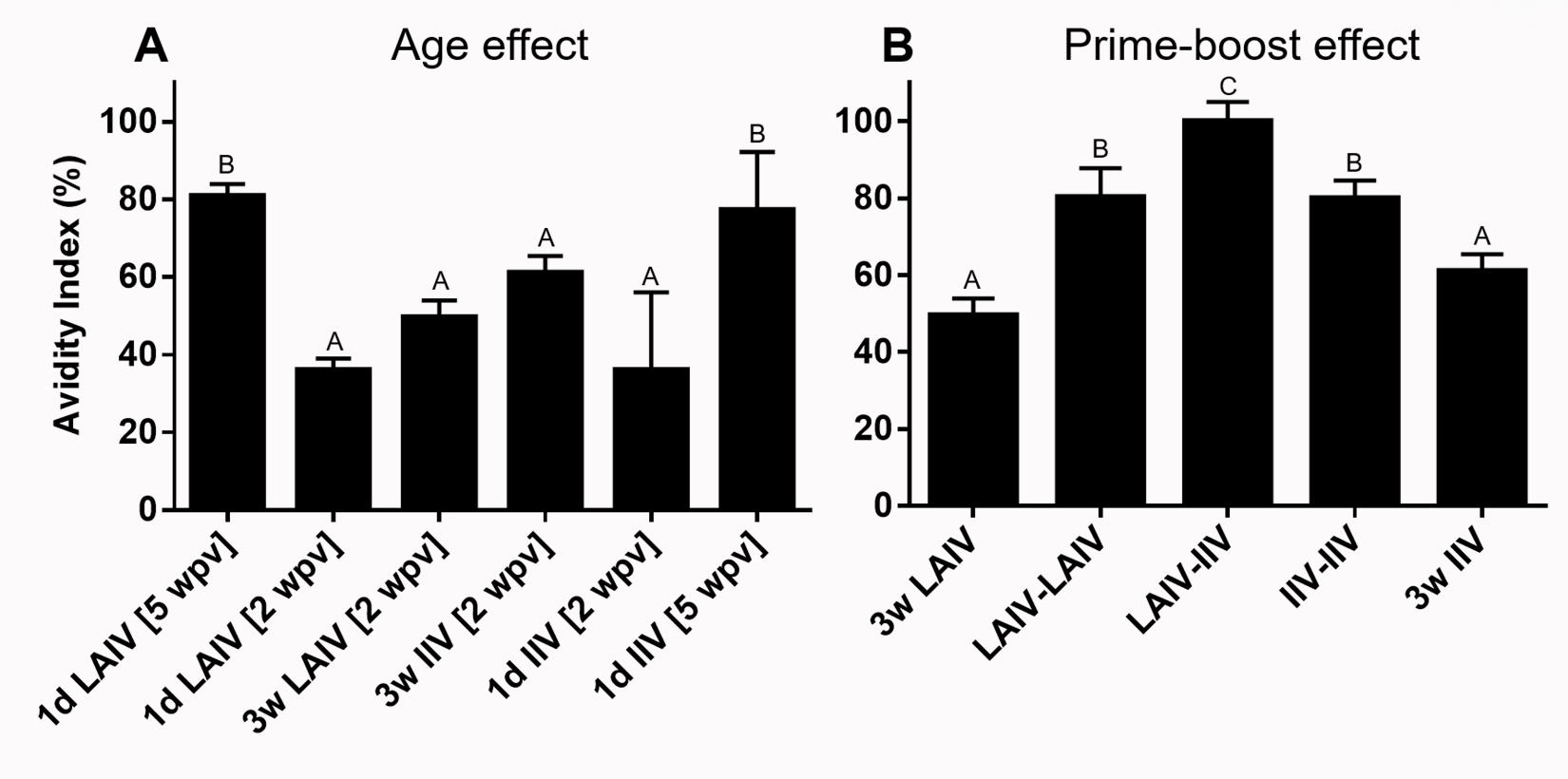**  **Fig 9. Effect of age and prime-boost vaccination on avidity index of serum HI antibodies.** Serum samples collected at 2 or 5 weeks post vaccination at 1 day of age or 2 weeks post boost-vaccination (5 weeks of age) were tested for their avidity as previously described [43]. (A) Age effect. (B) Prime-boost effect. Groups are arranged as described in Fig 5. Different letters inside the plot indicate significant differences between groups (p<0.05). | |  |
| - **Adverse events** | | **16** | No unexpected adverse events were observed and the experimental protocol was not modified. | |  |
| **DISCUSSION** | |  |  | |  |
| - **Interpretation/scientific implications** | | **17** | Many vaccine effectiveness studies have confirmed that LAIV has advantages over IIV in terms of stimulating naïve and developing immune systems [26, 27, 48]. Live vaccines can efficiently trigger the innate immune system, via diverse pathogen-associated molecular patterns, and lead to regulation of several host genes including type I IFNs [49]. The type I IFN is a crucial component of the innate antiviral immune system which regulates several hundred ISGs and shapes the development of adaptive immune responses [50-53]. Two genes, 2’,5’-OAS and Mx, are common among the ISGs induced by type I IFNs in chickens [32, 35]. Upregulation of ISGs by LAIV correlates well with rapid induction of adaptive immune responses and enhancement of protective efficacy in 4-week-old chickens [32]. We were interested in determining whether LAIV can also be efficacious in younger immunologically naïve chickens. The evidence provided in the current study indicates that LAIV can elicit higher levels of innate responses, mucosal IgA antibodies, and heterologous protection in 1-day-old chickens compared to IIV. In addition, a prime-boost vaccination regimen utilizing both live and inactivated vaccines was shown to elicit a robust activation of systemic and mucosal adaptive immune responses and provide superior protection from heterologous challenge virus. This regimen could be a big advantage in young birds which have limited numbers of functional antigen presenting cells [54, 55]. In addition to the general advantages of live vaccine, our LAIV is highly immunogenic via the action of truncated NS1 protein as proved in our previous studies [19, 32].  We previously showed that the ability of NS1-truncated LAIV candidates to induce a rapid development of adaptive immune responses and protective efficacy in 4 week old chickens was strongly associated with upregulation of 2′,5′-OAS and Mx genes [32]. A similar pattern of ISG expression was observed in birds vaccinated at 1 day of age, especially in trachea at 1-day post-vaccination (Fig 1), which suggests that the performance of LAIV is not diminished in young immunologically immature chickens. Induction of 2′,5′ OAS and Mx gene upregulation in trachea was also seen in the IIV group, but the magnitude was lower compared to the LAIV group (Fig 1). This difference in ISG upregulation corresponds well with the differences in the level of mucosal IgA, serum antibody cross-reactivity, and protective efficacy induced by each vaccine. ISG upregulation by LAIV did not correspond with upregulation of IFN gene transcription in birds vaccinated at 1 day of age (Fig 1), which is the same observation previously made in birds vaccinated at 4 weeks of age [32]. We plan to investigate further on this discrepancy in our future studies. It should be noted that oral administration of recombinant chicken IFN-α was shown to mimic LAIV by inducing a rapid serum antibody response to IIV vaccination in our previous study [32].  A single administration of LAIV in 1-day old chickens was sufficient to induce a significant increase in influenza virus-specific IgA antibodies in tears but failed to stimulate high serum antibody titers. Previous studies have repeatedly described serum IgG and mucosal IgA responses as key features that distinguish live vaccines from parenterally administered inactivated vaccines [56-58]. The protection afforded by IIV vaccination primarily depends on the action of neutralizing serum antibodies and the serum HI antibody titer is a strong indicator of IIV efficacy [24]. Serum antibody response does not always correlate with protective efficacy of live vaccines. The underlying mechanism of serum antibody-independent viral clearance by live vaccines is postulated to be driven by mucosal IgA or CD8+ and CD4+ T cell responses [24, 59-61]. This serum antibody-independent viral clearance can be utilized to enhance vaccine efficacy in young chickens considering their poor antibody production ability [62, 63]. According to a study of live infectious bronchitis (IB) virus vaccination in chickens of different ages, birds vaccinated immediately after hatching tended to produce lower levels of serum IgG antibodies that had lower avidity indices compared to the birds vaccinated at 4-weeks of age [36]. Our data is in total agreement with the IB study in that the avidity of anti-influenza virus IgG is age-dependent (Fig 9A). Further, the avidity of antibodies induced by vaccination at 1-day of age continued to increase as the bird’s immune system matured (Fig 9A). Furthermore, the age-dependent IgG avidity was not significantly different between the intranasally delivered LAIV and parenterally administered IIV (Fig 9) implying that avidity may not be influenced by the mode of antigen delivery. Although we did not measure the avidity of mucosal IgA in the current study, the IB study did not find a significant effect of age on IgA avidity [36].  Another study of live IB vaccination in 1-day-old chickens also showed an inefficient antibody response and the protective efficacy of the vaccine mostly correlated with induction of high levels of CD4+, CD8+ and IgA bearing B cells [64]. Also, the vaccination of 1 day old chickens with adenovirus vectored H5 and H7 influenza vaccine could induce IgA response in lachrymal fluid and increased interleukin-6 expression without inducing detectable levels of serum antibodies [65]. Therefore, our current findings are consistent with previous reports in that LAIV enhances mucosal IgA response and provides viral clearance that is less dependent on serum antibody response in young chickens.  There were clear differences between the protective efficacy of LAIV and IIV in 1-day-old chickens. It was surprising to see that IIV could provide partial protection (Fig 4) without detectable levels of pre-challenge heterologous HI antibodies (Fig 2) considering that IIV provides protection mainly via neutralizing serum antibodies [66]. It remains to be investigated why IIV vaccination transiently provided an almost complete block of virus replication at 3 dpc (Fig 4). Reduction of challenge virus by LAIV vaccination was apparent at 3 dpc and statistically significant at 5 dpc when most of the birds were completely protected (Fig 4). Lau *et al* [67] also observed a similar protection trend with cold-adapted LAIVs in mice challenged with a heterologous virus at 28 dpv. A significant reduction in challenge virus titer in lung was observed at 4 dpc, but not at 2 dpc [67]. The differences between the protective efficacy of LAIV and IIV in 1-day-old chickens may reflect differences in immunologic mechanisms. For the protection of young, immunologically immature birds with a limited capacity to produce sufficient levels of serum antibodies, we believe that the LAIV could be the better option. Additional optimization, such as using mucosal adjuvant or modification of vaccination regimen and dosage, may further improve protective efficacy of LAIV in 1-day-old chickens.  Considering that both vaccines could protect most of the birds at 5 dpc (Fig 4), each vaccine provided protection by different mechanisms. We reasoned that these vaccines could supplement or even synergize each other if used in a prime-boost regimen. We have provided data to prove the advantage of this approach. When 1-day-old chickens were intranasally primed with LAIV and subcutaneously boosted with IIV three weeks later (LAIV-IIV vaccination), they showed a rapid, robust, and highly cross-reactive serum antibody response (Figs 5 and 6, Table 3) with high avidity (Fig 9) and a high level of mucosal IgA response (Fig 7). The LAIV-IIV regimen was remarkably synergistic in enhancing cross-reactivity with the heterologous antigen. For example, the mean heterologous HI titers induced by LAIV-IIV were at least 7 times higher than the mean HI titers of 1d LAIV and 3w IIV groups combined (Fig 5). This kind of synergy between live and inactivated vaccines was previously described in humans. Talaat *et al* [28] found that the frequency and level of antibody response to inactivated H5N1 vaccination was significantly higher in subjects who were previously primed with homologous H5N1 LAIV. After prime vaccination, there was no detectable serum antibody response but the immune system was apparently sensitized to rapidly respond to IIV vaccination and produce high titers of broadly cross-reactive serum HI antibodies [28]. More recently, Pitisuttithum *et al* [28] have confirmed induction of high serum HI antibody titers by IIV in LAIV-experienced individuals and went further to demonstrate that the antibody boosting effect correlated strongly with an increase in circulating follicular T-helper cells and plasma B cells. It appears that the long-lasting priming effect of LAIV has no species barrier.  Using the concept of antigenic relatedness based on cross-HI test [45, 68], we have demonstrated that priming with LAIV leads to induction of antibodies with enhanced cross-reactivity to heterologous virus independently of whether the boosting vaccine is live or inactivated (Table 3, compare LAIV-LAIV and LAIV-IIV with the other groups). In addition, the antigenic relatedness was higher with serum produced by live CK/NJ/02 (H7N2) virus infection relative to the hyper-immune serum produced by a matched IIV (Table 3, compare the columns). Therefore, the antibodies produced by live viral infection seem to have a stronger cross-reactivity to heterologous antigen. However, the HI antibodies from single LAIV vaccinations did not show an enhanced heterologous cross-reactivity (Table 3) and we attribute this to poor antibody induction (Fig 5). Enhancement of serum antibody cross-reactivity by LAIV vaccination or live virus infection has been reported in previous studies. Jang *et al* [69] reported that antibodies induced by the 2009 H1N1 pandemic influenza vaccine were cross-reactive with seasonal H1 and H5 strains. Hancock *et al* [70] found that persons 60 years or older had serum antibodies that cross-reacted with the 2009 H1N1 pandemic virus while sera from younger adults or children were not cross-reactive. The authors speculated that the cross-reactive antibodies found in elderly individuals were a result of priming by natural infection with H1N1 virus followed by vaccination with swine-origin A/NJ/76 H1N1 vaccine [70]. While the mechanism involved in induction of cross-reactive antibodies remain to be investigated, our findings in the current study and the observations made in the above-mentioned studies [69, 70] suggest the potential of LAIV priming as a strategy that can be exploited to develop a broadly effective influenza vaccination regimen.  All three groups that received IIV at 3 weeks of age (LAIV-IIV, IIV-IIV, and 3w IIV) developed high levels of serum HI antibodies (Fig 5) and fully blocked heterologous challenge virus replication at 4 dpc (Fig 8). Thus, the effects of the immunological advantages of the LAIV-IIV prime-boost regimen (Figs 5, 6 and 7, Table 3) could not be resolved at the level of challenge virus replication because full protection was observed in all 3 groups (Fig 3). However, the LAIV-IIV regimen may be the best option under field settings where circulating strains can be much more distantly related than the challenge virus used in this study.  **Conclusions**  One of the perceived risks of vaccinating commercial poultry against influenza is the possibility of the vaccine protecting birds from disease without preventing replication and spread of the virus. We have demonstrated that a single dose of LAIV is able to induce stronger innate and mucosal IgA responses and protect young immunologically immature chickens better than a single dose of conventional IIV. Most importantly, priming with LAIV led to a synergistic serum antibody induction by IIV and enhancement of antibody cross-reactivity, thereby increasing the chance of protection from distantly related strains. Our prime-boost vaccine strategy requires further improvements to address possible limitations such as the cost effectiveness and safety issues. In addition, we are looking forward to evaluating the efficacy of the prime-boost regimen against different strains of HPAI viruses. | |  |
| - **Generalizability/translation** | | **18** | One of the perceived risks of vaccinating commercial poultry against influenza is the possibility of the vaccine protecting birds from disease without preventing replication and spread of the virus. We have demonstrated that a single dose pc4-LAIV is able to induce stronger innate and mucosal IgA responses and protect young, immunologically immature, chickens better than a single dose of conventional IV. Most importantly, priming with pc4-LAIV led to a synergistic serum antibody induction by IV and enhancement of antibody cross-reactivity, thereby increasing the chance of protection from distantly related strains. This vaccination regimen warrants further optimization and investigation on its protective efficacy against heterosubtypic challenge.  Our data study has opened a new ground with potential to help further development of highly efficacious LAIV for chickens: An in-depth study such as systems biology is required to determine which ISGs need to be upregulated to enhance *NS1-truncated* LAIV efficacy. | |  |
| - **Funding** | | **19** | This study was partially supported by Agriculture and Food Research Initiative Competitive Grant no. 2013-67015-20476 from the USDA National Institute of Food and Agriculture; Doctoral SEEDS Grant no. OHOA1006 from the Ohio Agricultural Research and Development Center. | |  |

1. Capua I, Mutinelli F, Pozza MD, Donatelli I, Puzelli S, Cancellotti FM. The 1999-2000 avian influenza (H7N1) epidemic in Italy: veterinary and human health implications. Acta Trop. 2002;83(1):7-11. PMID: 12062787.

2. Basuno E, Yusdja Y, Ilham N. Socio-economic impacts of avian influenza outbreaks on small-scale producers in Indonesia. Transbound Emerg Dis. 2010;57(1-2):7-10. PMID: 20537092.

3. Zhu W, Wang C, Wang BZ. From Variation of Influenza Viral Proteins to Vaccine Development. Int J Mol Sci. 2017;18(7). PMCID: PMCPMC5536042.

4. Alexander DJ. An overview of the epidemiology of avian influenza. Vaccine. 2007;25(30):5637-44. Epub 2006/11/28. doi: 10.1016/j.vaccine.2006.10.051. PMID: 17126960.

5. CDC. Avian Influenza Current Situation Summary [updated April 12, 2017; cited 2017 August 28]. Available from: <https://www.cdc.gov/flu/avianflu/avian-flu-summary.htm>.

6. USDA. Avian influenza [cited 2017 August 28]. Available from: <https://www.usda.gov/topics/animals/one-health/avian-influenza>.

7. Hsu SM, Chen TH, Wang CH. Efficacy of avian influenza vaccine in poultry: a meta-analysis. Avian Dis. 2010;54(4):1197-209. PMID: 21313840.

8. FAO. The Global Strategy for Prevention and Control of H5N1 Highly Pathogenic Avian Influenza. Rome, Italy2007.

9. Lee CW, Suarez DL. Avian influenza virus: prospects for prevention and control by vaccination. Anim Health Res Rev. 2005;6(1):1-15. PMID: 16164006.

10. Capua I, Marangon S. Control of avian influenza in poultry. Emerg Infect Dis. 2006;12(9):1319-24. doi: 10.3201/eid1209.060430. PMID: 17073078.

11. Nations FaAOotU. Avian Influenza: Questions & Answers. 7. How is avian influenza transmitted? 2017 [cited 2017 August 28]. Available from: <http://www.fao.org/avianflu/en/qanda.html>.

12. USDA. 2017 Highly Pathogenic Avian Influenza: USDA; 2017 [cited 2017 September 25th]. Available from: <https://www.aphis.usda.gov/aphis/ourfocus/animalhealth/animal-disease-information/avian-influenza-disease/defend-the-flock/2017-hpai>.

13. Swayne DE. Avian influenza vaccines and therapies for poultry. Comp Immunol Microbiol Infect Dis. 2009;32(4):351-63. PMID: 18442853.

14. Suarez DL. Avian influenza: our current understanding. Anim Health Res Rev. 2010;11.

15. Doherty PC, Turner SJ, Webby RG, Thomas PG. Influenza and the challenge for immunology. Nat Immunol. 2006;7(5):449-55. PMID: 16622432.

16. Suarez DL, Pantin-Jackwood MJ. Recombinant viral-vectored vaccines for the control of avian influenza in poultry. Veterinary Microbiology. 2017;206:144-51.

17. Suarez DL, Lee CW, Swayne DE. Avian influenza vaccination in North America: strategies and difficulties. Dev Biol (Basel). 2006;124:117-24. PMID: 16447502.

18. Wang L, Yassine H, Saif YM, Lee CW. Developing live attenuated avian influenza virus in ovo vaccines for poultry. Avian Dis. 2010;54(1 Suppl):297-301. PMID: 20521649.

19. Wang L, Suarez DL, Pantin-Jackwood M, Mibayashi M, Garcia-Sastre A, Saif YM, et al. Characterization of influenza virus variants with different sizes of the non-structural (NS) genes and their potential as a live influenza vaccine in poultry. Vaccine. 2008;26(29-30):3580-6. PMID: 18539366

20. Richt JA, Garcia-Sastre A. Attenuated influenza virus vaccines with modified NS1 proteins. Curr Top Microbiol Immunol. 2009;333:177-95. PMID: 19768406.

21. Rice JT, Bierer BW, Dick JW. Vaccination of chickens through the drinking water with a live, Pasteurella multocida, vaccine. Poult Sci. 1979;58(1):18-22. Epub 1979/01/01. PubMed PMID: 471883.

22. Toro H, Tang DC. Protection of chickens against avian influenza with nonreplicating adenovirus-vectored vaccine. Poult Sci. 2009;88(4):867-71. PMID: 19276437.

23. Swayne DE, Beck JR, Kinney N. Failure of a recombinant fowl poxvirus vaccine containing an avian influenza hemagglutinin gene to provide consistent protection against influenza in chickens preimmunized with a fowl pox vaccine. Avian Dis. 2000;44(1):132-7. PMID: 10737653.

24. Sridhar S, Brokstad KA, Cox RJ. Influenza Vaccination Strategies: Comparing Inactivated and Live Attenuated Influenza Vaccines. In: Gilbert S, editor. Vaccines (Basel). 32015. p. 373-89.

25. He XS, Holmes TH, Zhang C, Mahmood K, Kemble GW, Lewis DB, et al. Cellular immune responses in children and adults receiving inactivated or live attenuated influenza vaccines. J Virol. 2006;80(23):11756-66. PMID: 16971435

26. Hoft DF, Babusis E, Worku S, Spencer CT, Lottenbach K, Truscott SM, et al. Live and inactivated influenza vaccines induce similar humoral responses, but only live vaccines induce diverse T-cell responses in young children. J Infect Dis. 2011;204(6):845-53. PMID: 21846636

27. Ambrose CS, Wu X, Knuf M, Wutzler P. The efficacy of intranasal live attenuated influenza vaccine in children 2 through 17 years of age: A meta-analysis of 8 randomized controlled studies. Vaccine. 2012;30(5):886-92.

28. Talaat KR, Luke CJ, Khurana S, Manischewitz J, King LR, McMahon BA, et al. A live attenuated influenza A(H5N1) vaccine induces long-term immunity in the absence of a primary antibody response. J Infect Dis. 2014;209(12):1860-9. PMID: 24604819

29. Pitisuttithum P, Boonnak K, Chamnanchanunt S, Puthavathana P, Luvira V, Lerdsamran H, et al. Safety and immunogenicity of a live attenuated influenza H5 candidate vaccine strain A/17/turkey/Turkey/05/133 H5N2 and its priming effects for potential pre-pandemic use: a randomised, double-blind, placebo-controlled trial. Lancet Infect Dis. 2017;17(8):833-42. PMID: 28533093

30. Wu D, Zou S, Bai T, Li J, Zhao X, Yang L, et al. Poultry farms as a source of avian influenza A (H7N9) virus reassortment and human infection. Sci Rep. 2015;5:7630. PMID: 25591105

31. Vijaykrishna D, Bahl J, Riley S, Duan L, Zhang JX, Chen H, et al. Evolutionary dynamics and emergence of panzootic H5N1 influenza viruses. PLoS Pathog. 2008;4(9):e1000161. PMID: 18818732

32. Jang H, Ngunjiri JM, Lee CW. Association between Interferon Response and Protective Efficacy of NS1-Truncated Mutants as Influenza Vaccine Candidates in Chickens. PLoS One. 2016;11(6):e0156603. PMID: 27257989

33. Marcus PI, Ngunjiri JM, Sekellick MJ, Wang L, Lee CW. In vitro analysis of virus particle subpopulations in candidate live-attenuated influenza vaccines distinguishes effective from ineffective vaccines. J Virol. 2010;84(21):10974-81. PMID: 20739541

34. Talon J, Salvatore M, O'Neill RE, Nakaya Y, Zheng H, Muster T, et al. Influenza A and B viruses expressing altered NS1 proteins: A vaccine approach. Proc Natl Acad Sci USA. 2000;97.

35. Meng S, Yang L, Xu C, Qin Z, Xu H, Wang Y, et al. Recombinant chicken interferon-alpha inhibits H9N2 avian influenza virus replication in vivo by oral administration. J Interferon Cytokine Res. 2011;31(7):533-8. PMID: 21323426.

36. van Ginkel FW, Padgett J, Martinez-Romero G, Miller MS, Joiner KS, Gulley SL. Age-dependent immune responses and immune protection after avian coronavirus vaccination. Vaccine. 2015;33(23):2655-61. PMID: 25910920.

37. Pawar SD, Murtadak VB, Kale SD, Shinde PV, Parkhi SS. Evaluation of different inactivation methods for high and low pathogenic avian influenza viruses in egg-fluids for antigen preparation. J Virol Methods. 2015;222:28-33. PMID: 25997377.

38. Elaish M, Ngunjiri JM, Ali A, Xia M, Ibrahim M, Jang H, et al. Supplementation of inactivated influenza vaccine with norovirus P particle-M2e chimeric vaccine enhances protection against heterologous virus challenge in chickens. PLoS One. 2017;12(2):e0171174. PMID: 28151964

39. Ngunjiri JM, Ali A, Boyaka P, Marcus PI, Lee CW. In Vivo Assessment of NS1-Truncated Influenza Virus with a Novel SLSYSINWRH Motif as a Self-Adjuvanting Live Attenuated Vaccine. PLoS One. 2015;10(3):e0118934. PMID: 25790187

40. Spackman E, Senne DA, Myers TJ, Bulaga LL, Garber LP, Perdue ML, et al. Development of a real-time reverse transcriptase PCR assay for type A influenza virus and the avian H5 and H7 hemagglutinin subtypes. J Clin Microbiol. 2002;40(9):3256-60. PMID: 12202562

41. Lee CW, Suarez DL. Application of real-time RT-PCR for the quantitation and competitive replication study of H5 and H7 subtype avian influenza virus. J Virol Methods. 2004;119(2):151-8. PMID: 15158597.

42. Jang H, Koo BS, Jeon EO, Lee HR, Lee SM, Mo IP. Altered pro-inflammatory cytokine mRNA levels in chickens infected with infectious bronchitis virus. Poult Sci. 2013;92(9):2290-8. PMID: 23960111.

43. Kapczynski DR, Jiang HJ, Kogut MH. Characterization of cytokine expression induced by avian influenza virus infection with real-time RT-PCR. Methods Mol Biol. 2014;1161:217-33. PMID: 24899432.

44. (OIE) WOfAH. Manual of Diagnostic Tests and Vaccines for Terrestrial Animals 2012 [cited 2017 August 28]. Available from: <http://www.oie.int/manual-of-diagnostic-tests-and-vaccines-for-terrestrial-animals/>.

45. Archetti I, Horsfall FL, Jr. Persistent antigenic variation of influenza A viruses after incomplete neutralization in ovo with heterologous immune serum. J Exp Med. 1950;92(5):441-62. PMID: 14778924

46. Ganapathy K, Cargill PW, Jones RC. A comparison of methods of inducing lachrymation and tear collection in chickens for detection of virus-specific immuoglobulins after infection with infectious bronchitis virus. Avian Pathol. 2005;34(3):248-51. PMID: 16191709.

47. To KK, Zhang AJ, Hung IF, Xu T, Ip WC, Wong RT, et al. High titer and avidity of nonneutralizing antibodies against influenza vaccine antigen are associated with severe influenza. Clin Vaccine Immunol. 2012;19(7):1012-8. PMID: 22573737

48. Forrest BD, Pride MW, Dunning AJ, Capeding MR, Chotpitayasunondh T, Tam JS, et al. Correlation of cellular immune responses with protection against culture-confirmed influenza virus in young children. Clin Vaccine Immunol. 2008;15(7):1042-53. PMID: 18448618

49. Iwasaki A, Pillai PS. Innate immunity to influenza virus infection. Nat Rev Immunol. 2014;14(5):315-28. doi: 10.1038/nri3665. PMID: 24762827

50. Schneider WM, Chevillotte MD, Rice CM. Interferon-Stimulated Genes: A Complex Web of Host Defenses. Annu Rev Immunol. 2014;32:513-45. PMID: 24555472

51. McNab F, Mayer-Barber K, Sher A, Wack A, O'Garra A. Type I interferons in infectious disease. Nat Rev Immunol. 2015;15(2):87-103.

52. Garcia-Sastre A. Induction and evasion of type I interferon responses by influenza viruses. Virus Res. 2011;162(1-2):12-8. PMID: 22027189

53. Schoggins JW, Wilson SJ, Panis M, Murphy MY, Jones CT, Bieniasz P, et al. A diverse range of gene products are effectors of the type I interferon antiviral response. Nature. 2011;472(7344):481-5. PMID: 21478870

54. Demirjian A, Levy O. Safety and Efficacy of Neonatal Vaccination. Eur J Immunol. 2009;39(1):36-46. PMID: 19089811.

55. Holmgren J, Czerkinsky C. Mucosal immunity and vaccines. Nat Med. 2005; 11(4 Suppl):S45-53. PMID:15812489

56. Ghendon Y. The immune response to influenza vaccines. Acta Virol. 1990;34(3):295-304. PMID: 1980401.

57. Barría MI, Garrido JL, Stein C, Scher E, Ge Y, Engel SM, et al. Localized Mucosal Response to Intranasal Live Attenuated Influenza Vaccine in Adults. The Journal of Infectious Diseases. 2013;207(1):115-24..

58. Hoft DF, Lottenbach KR, Blazevic A, Turan A, Blevins TP, Pacatte TP, et al. Comparisons of the Humoral and Cellular Immune Responses Induced by Live Attenuated Influenza Vaccine (LAIV) and Inactivated Influenza Vaccine (IIV) in Adults. Clinical and Vaccine Immunology. 2016; 24:e00414-16. PMID:27847366

59. Brown DM, Dilzer AM, Meents DL, Swain SL. CD4 T cell-mediated protection from lethal influenza: perforin and antibody-mediated mechanisms give a one-two punch. J Immunol. 2006;177(5):2888-98. PMID: 16920924.

60. Topham DJ, Tripp RA, Sarawar SR, Sangster MY, Doherty PC. Immune CD4+ T cells promote the clearance of influenza virus from major histocompatibility complex class II -/- respiratory epithelium. J Virol. 1996;70(2):1288-91. PMID: 8551597

61. Chen GL, Lau YF, Lamirande EW, McCall AW, Subbarao K. Seasonal influenza infection and live vaccine prime for a response to the 2009 pandemic H1N1 vaccine. Proc Natl Acad Sci U S A. 2011;108:1140-5. PMID: 21199945

62. Frommer A, Freidlin PJ, Bock RR, Leitner G, Chaffer M, Heller ED. Experimental vaccination of young chickens with a live, non‐pathogenic strain of Escherichia coli. Avian Pathology. 1994;23(3):425-33. PMID: 18671110

63. Starck JM, Ricklefs RE. Avian Growth and Development: Evolution Within the Altricial-precocial Spectrum: Oxford University Press; 1998.

64. Awad F, Hutton S, Forrester A, Baylis M, Ganapathy K. Heterologous live infectious bronchitis virus vaccination in day-old commercial broiler chicks: clinical signs, ciliary health, immune responses and protection against variant infectious bronchitis viruses. Avian Pathol. 2016;45(2):169-77. PMID: 26743315.

65. Toro H, van Ginkel FW, Tang DC, Schemera B, Rodning S, Newton J. Avian Influenza Vaccination in Chickens and Pigs with Replication-Competent Adenovirus–Free Human Recombinant Adenovirus 5. Avian Dis. 2010;54(1 Suppl):224-31. PMID: 20521636

66. Gomez Lorenzo MM, Fenton MJ. Immunobiology of Influenza Vaccines. Chest. 2013;143(2):502-10. PMID: PMC3619640.

67. Lau YF, Santos C, Torres-Vélez FJ, Subbarao K. The Magnitude of Local Immunity in the Lungs of Mice Induced by Live Attenuated Influenza Vaccines Is Determined by Local Viral Replication and Induction of Cytokines. J Virol. 2011;85: 76-85.

68. Lee MS, Chen JSE. Predicting Antigenic Variants of Influenza A/H3N2 Viruses. Emerg Infect Dis. 2004;10:1385-90. PMCID:PMC3320420

69. Jang YH, Byun YH, Lee YJ, Lee YH, Lee K-H, Seong BL. Cold-adapted pandemic 2009 H1N1 influenza virus live vaccine elicits cross-reactive immune responses against seasonal and H5 influenza A viruses. Journal of Virology. 2012;86(10):5953-8. PMID: 22438541.

70. Hancock K, Veguilla V, Lu X, Zhong W, Butler EN, Sun H, et al. Cross-Reactive Antibody Responses to the 2009 Pandemic H1N1 Influenza Virus. New England Journal of Medicine. 2009;361(20):1945-52.
